# Physiologically Based Pharmacokinetic Modeling in Neonates: Current Status and Future Perspectives

**DOI:** 10.3390/pharmaceutics15122765

**Published:** 2023-12-12

**Authors:** Wei Zhang, Qian Zhang, Zhihai Cao, Liang Zheng, Wei Hu

**Affiliations:** Department of Clinical Pharmacology, The Second Affiliated Hospital of Anhui Medical University, Hefei 230601, China; mathdrug02@gmail.com (W.Z.); vivian8011@163.com (Q.Z.); caozh0826@163.com (Z.C.)

**Keywords:** PBPK, modeling and simulation, term infants, preterm infants

## Abstract

Rational drug use in special populations is a clinical problem that doctors and pharma-cists must consider seriously. Neonates are the most physiologically immature and vulnerable to drug dosing. There is a pronounced difference in the anatomical and physiological profiles be-tween neonates and older people, affecting the absorption, distribution, metabolism, and excretion of drugs in vivo, ultimately leading to changes in drug concentration. Thus, dose adjustments in neonates are necessary to achieve adequate therapeutic concentrations and avoid drug toxicity. Over the past few decades, modeling and simulation techniques, especially physiologically based pharmacokinetic (PBPK) modeling, have been increasingly used in pediatric drug development and clinical therapy. This rigorously designed and verified model can effectively compensate for the deficiencies of clinical trials in neonates, provide a valuable reference for clinical research design, and even replace some clinical trials to predict drug plasma concentrations in newborns. This review introduces previous findings regarding age-dependent physiological changes and pathological factors affecting neonatal pharmacokinetics, along with their research means. The application of PBPK modeling in neonatal pharmacokinetic studies of various medications is also reviewed. Based on this, we propose future perspectives on neonatal PBPK modeling and hope for its broader application.

## 1. Introduction

The lack of pediatric drug research is due to difficulties in recruiting a sufficient number of children for clinical studies, ethical concerns associated with enrolling younger children, low consent rates from neonatal parents, limited blood volume availability, and high costs [1]. Consequently, fewer drugs have been authorized and are available for pediatric clinical therapy as compared to the drugs available for adults.

Children are not simply smaller adults because the difference between them is not only due to body weight or surface area but also due to physiological and biochemical changes according to age, particularly in early life [2,3]. Childhood is characterized by pronounced differences in growth and development [4]. Compared to other pediatric subpopulations, the weight of infants from term birth to two years of age increases dramatically by approximately three times, and premature neonates can triple their weight several weeks after receiving postnatal care [5]. Growth rates are most prominent during the last trimester of pregnancy and the first trimester after delivery and are much higher than the pubertal growth spurt [6]. Additionally, maturational physiological changes in structural and functional development include the development of organ system functions, cardiac output (CO), organ perfusion, permeability, glomerular filtration rate (GFR), and the ontogeny of metabolic enzymes and transport [7]. Physiological factors in specific neonatal subpopulations differ significantly from those in general adult and pediatric populations, resulting in alterations to the main pharmacokinetic (PK) processes, including absorption, distribution, metabolism, and excretion (ADME). For example, increased gastric pH and emptying time and decreased gastrointestinal size and transport time are known to reduce the absorption rate of biopharmaceutical classification system class I and II compounds in preterm neonates [8,9,10]. The reported lower plasma protein concentration, higher body water content, and lower blood volume affect drug distribution in neonates [5]. Studies also have shown age-related changes in the activity and expression of uridine-5-diphosphate glucuronosyltransferases (UGTs) and cytochrome p450 (CYP450) enzymes, resulting in reduced drug clearance [11,12]. Neonatal renal clearance systems, such as GFR, glomerular permeability, and reabsorption, are immature and change rapidly throughout the first few months of life, reducing drug excretion [13]. These growth and development processes are not linear and display interindividual variation [14]. However, there are still many knowledge gaps regarding the ontogeny of the organs or tissues involved in ADME processes, the effects of diseases on physiological maturation, and the influence of developmental factors on drug disposition and pharmacological effects. Therefore, one of the greatest difficulties in pediatric drug therapy is constantly exploring and adjusting the proper, safe, and effective dose for neonates using evidence-based information rather than empirical treatment.

Traditionally, allometric principles based on age, body weight, and body surface area have been widely used to extrapolate suitable doses for children from adult data. Using the power function, allometric scaling can reflect the relation between PK parameters, such as clearance, volume of distribution, half-life, and size for different ages, from preterm neonates to adults [15,16,17,18,19,20]. Previously, the allometric exponent used for all age groups was fixed as 0.75 [21,22]. At present, this exponent is challenged for younger children (<2 years of age), and the emerging age-dependent exponent model improved predictive performance by applying 1.2 as the allometric exponent for preterm, 1.1 for term neonates (≤3 months of age), and 1.0 for children > 3 months to 2 years [15]. However, allometry scaling cannot accurately describe the developmental changes in the drug clearance system, particularly drug-metabolizing enzymes, as well as the relation between the concentration effect and response, which may result in misestimation of drug kinetics, imprecise dosing of drugs, drug-induced toxicity, or reduced efficacy in younger children [23].

Currently, modeling and simulation approaches, including population pharmacokinetic (popPK) and physiologically based pharmacokinetic (PBPK) models, have emerged as cornerstones of pediatric drug development and rational prescription for pediatric subpopulations [24]. PopPK modeling integrates retrospective studies of pooled clinical data with allometric and maturation functions, quantitatively describes PK variability within groups, and predicts drug concentration in vivo for other doses and age ranges. Moreover, covariate analysis can explain the sources of PK variability, such as physical characteristics, lifestyle habits, and biochemical indicators, and can estimate the extent to which patient-specific covariates affect variability. However, popPK modeling requires sufficient sampling of a population and knowledge of appropriate allometric and maturation functions, and PK prediction is limited to the scaling of selected parameters within the population and dose studied. Compared with popPK modeling, the PBPK technique integrates time-dependent physiological growth and organ function maturation with physiochemical drug properties using mathematical modeling, enabling prospective prediction of the PK behavior of drugs across the pediatric age span [25,26]. In general, various organs or tissue compartments mimicking the actual anatomy of the body are interconnected by blood circulation loops in PBPK modeling, and the direction of blood flow does not differ from that of the real human body. In this system, drugs are carried by the bloodstream and distributed in a dozen organs and tissues through passive diffusion. The involvement of drug disposition proteins, such as metabolic enzymes and transporters, has also been described. Subsequently, the concentration-versus-time profiles of drugs in the plasma, specific organs, or tissues are predicted using mass balance equations [27,28,29]. Over the past few decades, there has been a better understanding of developmental changes (growth and maturation) in ADME of the neonatal population. Previous studies also showed that PBPK modeling provided better predictions of clearance or exposure in children than empirical modeling, especially in children < 2 years of age [21,30]. Thus, neonatal PBPK modeling can maximize preclinical and adult/child clinical information and can be extrapolated between different populations to fill the knowledge gap in neonatal drug development. Moreover, regulatory authorities have encouraged the use of validated nonclinical research methods for dose selection and optimization in the pediatric population [22]. In this review, we aimed to illustrate the (patho) physiological changes that affect the pharmacokinetic properties of preterm and term infants, the current status and challenges of neonatal PBPK modeling, and how pediatricians make clinical decisions through PBPK modeling and simulation.

## 2. Methods

Firstly, we searched the information related to physiological changes from fetuses to adults, and compared the physiological difference between preterm and term infants. We also investigated pathological conditions that affect the PK parameters of neonates. To investigate the current status and application of neonatal PBPK simulation, we then performed a systematic review according to the Preferred Reporting Items for Systematic Reviews and Meta-Analyses (PRISMA) guidelines [31].

### 2.1. Literature Search

We conducted a systematic search of the PubMed, Web of Science, and Google Scholar databases up to 7 September 2023. Neonatal PBPK modeling of commonly used drugs were potentially eligible for this review. The search strategy included the medical subject headings (MeSH) and text words as: [“Physiologically based pharmacokinetic modeling” OR “Physiologically-based pharmacokinetic modeling” OR “PBPK”] AND [“Infant*” OR “Newborn*” OR “New-born” OR “Neonate*” OR “Neonatal”]. 

### 2.2. Inclusion and Exclusion Criteria

Original articles published in journals were eligible for this systematic review if they included neonatal PBPK modeling. Other publications such as reviews, comparative studies, animal modeling, toxicology and environmental science modeling, placental or lactational transfer modeling, popPK modeling, drug–drug interaction, and pediatrics not including newborns were excluded.

### 2.3. Article Selection

Two authors (WZ and QZ) independently performed the initial search and screened titles and abstracts for relevance. Full texts of potentially eligible articles were retrieved and read based on inclusion and exclusion criteria. The final articles included in this manuscript were decided after discussion among all the authors. 

### 2.4. Neonatal PBPK Modeling Mechanism and Workflow

**Figure 1:**
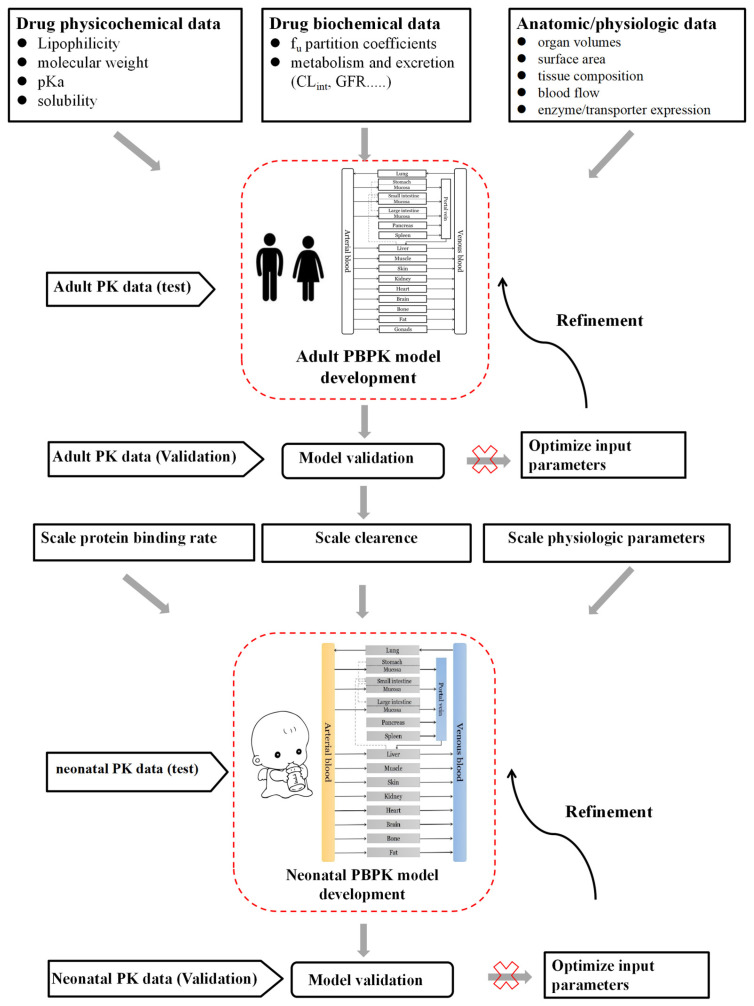
Schematic representing the neonatal physiologically based pharmacokinetic (PBPK) modeling workflow.

PBPK modeling and simulation are valuable techniques for developing pediatric drugs for dose selection, early clinical trial design, correlation with target organ toxicities, possible drug–drug interaction, and the effect of impaired organ function on pharmacokinetic parameters. Both whole-body and simplified mechanistic lumping PBPK models were built to combine the specific physicochemical properties of drugs and anatomic and physiological information. Modifying the adult PBPK model is the standard method for creating a neonatal PBPK model. This strategy is based on the assumption that clearance routes in adults and neonates (preterm and term) are comparable, and that the structure of the PBPK simulation is similar in adults and newborns [32]. The process of neonatal PBPK modeling is shown in Figure 1. The first step was to simulate the pharmacokinetic profiles of the adult population. By evaluating this adult PBPK model through comparison with observed data in actual practice, the PBPK model was refined if any inaccurate forecast by the adult PBPK model was found. After verifying the adult PBPK model, preterm and full-term neonatal PBPK models were developed using relevant age-dependent physiological changes. This simulation was run to obtain plasma drug concentration profiles and further validated using sparse clinical data from neonates. We should also note that PBPK modeling should reflect observably growing physiology with neonates’ postnatal days, particularly when simulating multiple dosing scenarios in this population, as implemented in some dedicated modeling tools like PK-Sim^®^ [33].

## 3. Results

### 3.1. Neonatal Physiologic Changes Affecting the Drug Disposition Process

#### 3.1.1. Drug Absorption

Absorption describes the concentration–time pattern when administered through nonvascular routes, such as oral, intramuscular, percutaneous, intranasal, and rectal routes, and is usually expressed as bioavailability and absorption rate. Suppose drugs are administered intramuscularly. In this case, the absorption rate of neonates is challenging to estimate because skeletal muscle blood flow and muscular contraction are reported to be reduced, and the body water content and capillary density in skeletal muscles are reported to be much higher in neonates [34,35]. The common drugs administrated intramuscularly for neonates are vitamin K formulations and vaccines. The extent of transdermal formulation absorption is inversely correlated to the thickness of epidermis and directly related to the degree of skin hydration and skin surface area to body weight ratio [35]. The epidermal development is dependent on gestational age (GA) and is complete at the 34th gestational week (GW) [36]. The full-term neonates have intact skin barrier function while preterm infants lack the vernix caseosa and are more sensitive to percutaneous toxicity [36]. Hence, percutaneous administration of drugs must be conservative in the first few weeks of life. Rectal administration is a good alternative in emergency situations when neonates are unconscious, uncooperative, or vomiting. Due to the fact that rectal administration is a less invasive route, it is well accepted in infants. However, the high variability of drug exposure in neonates limits the use of rectal administration, although this route can avoid first-pass metabolism. Previous study has showed that the absorption rate and extent of rectal acetaminophen are lower than oral administration in preterm and term neonates [37]. Most full-term newborns have entered the alveolar development stage after birth, whereas highly preterm infants are at the bronchiole and alveolar epithelial development stage and late preterm infants are at the saccular phase of lung development, so the inhalation routine requires caution in premature neonates [38,39]. The rate and extent of oral drug absorption in neonates are affected by a variety of physiological factors, including gastric pH and emptying time, characteristics of gastric juices, small and large intestinal transit time, gastric and intestinal volume, bile production, intestinal membrane transporters, and drug-metabolizing enzymes [40]. 

Oral administration is the preferred route for pediatric patients, and liquid dosage forms are preferred in neonates because they are easy to administer. The primary sites of oral drug absorption in newborns are the stomach, small intestine, and colon [41]. 

The structural and functional maturity of the neonatal gastrointestinal tract varies, mainly depending on the term or preterm birth. Drug absorption in the gut is mainly affected by the gastric pH and gastric emptying time. The gastric pH of the neonate drops from approximately 7 to approximately 2 after birth, then rises to above 4 and finally declines back to approximately 2 in two years [42]. Moreover, the gastric pH in preterm neonates is relatively higher than term infants due to lower levels of basal acid and gastric secretions [13]. A relatively high gastric pH in the body may increase absorption of weakly alkaline drugs and reduce absorption of weakly acidic drugs. Gastric motility is low in highly preterm newborns and approaches full-term newborns after 32 weeks of gestation, whereas gastric emptying in neonates is slower than that in older children [43]. Anatomical differentiation of the human gut reaches neonatal levels by 20 weeks of gestation, and functional maturation, such as digestive enzyme secretion or closure of tight junctions, develops at different rates and generally occurs after the GW of 32–34 [44,45]. A previous study showed that small and large intestinal lengths increase from fetal to maturation age [46]. Low production of bile acids and bile salts in the intestinal lumen at birth may affect enterohepatic bile circulation; however, passive reuptake and active transport of bile are also present [47]. Intestinal motility affects intestinal drug absorption by changing the intestinal transit time. An in vitro model suggested that, similar to adults, the intestinal transit time in term neonates was approximately four hours, whereas that in preterm newborns was approximately four-fold longer due to reduced intestinal motility and peristalsis [48]. Specifically, the overall absorption is delayed and incomplete in newborns. The expression of intestinal P-glycoprotein in neonates is low, especially in those born before the 28th GW [49]. Similarly, the protein expression of CYP3A4 is limited after birth and increases from neonates to adults, which may result in an increase in bioavailability in newborns [46,50]. Drug absorption in the gastrointestinal tract is also affected by regional blood flow, especially in critical ill status such as hypoxia. In addition, pancreatic and biliary functions are immature after birth and develop with age. The physiologic changes affecting oral drug absorption in neonates are listed in Table 1.

#### 3.1.2. Drug Distribution

Once a drug enters the systemic circulation, it is distributed to various tissues and organs, which is important for the interaction between drugs and targets. Drug distribution involves passive processes, such as protein-binding permeability, and active processes, such as influx and efflux through transporters [47]. Age-related changes in the body composition and function of the cardiovascular system, compound properties, such as lipophilicity, protein binding, and disease status affect drug distribution in vivo [47]. An impressive increase in body weight, length, and surface area has been observed during early life (Table 2). In addition to the changes mentioned above, changes in body composition were predominant in newborns. Water is the major component of cells and tissues, being responsible for about 60–65% of an adult’s weight, and a higher percentage in full-term (80–85%) and preterm infants (90%) [61,62,63]. The contraction of extracellular water with age results in a 5–7% weight loss in term newborns and a higher weight loss of 10–15% in preterm neonates with meager birth weight (<1500 g) at the end of the first week [61]. Combined with the low body fat percentage at birth (10–15%), neonates require larger doses of hydrophilic drugs to achieve similar efficacy because the ratio of water to lipids is higher in newborns [62]. Both these factors are more significant in the preterm infants, resulting in lower drug plasma concentrations in these different compartments using a body weight-based dosing mode [63,64,65]. The amount and type of plasma proteins determine drug distribution and action, because only unbound drugs can be distributed in vivo and exert pharmacological effects by binding to the corresponding receptors [66]. Human serum albumin (HSA) generally binds to acidic exogenous compounds, whereas alpha 1-glycoprotein (AAG) has a high affinity for basic lipophilic compounds. Previous research reported that the concentration of HSA increased until 20 years of age, then started decreasing with age [67]. Preterm neonates have lower serum albumin concentrations (2.36 g/dL in preterm infants born at 23–34 weeks) compared to term babies (3.43 g/dL) for at least the first three months of life [68]. AAG concentrations seem to stay stable at a low level until 260 days of GA and then begin to rise significantly [69]. Therefore, for highly protein-bound drugs, the amount of free drug and the related pharmacological effect in the body appears to increase in preterm and full-term infants. In addition, physiologically elevated endogenous enzymes, such as bilirubin or fatty acids, competitively bind to plasma proteins, resulting in drug displacement and increased unbound drug concentration [70,71]. Diseases, such as cardiogenic shock or patent ductus arteriosus, may affect CO, regional blood flow, or tissue permeability, thereby increasing the volume of distribution and requiring a higher dose of drugs to achieve a sufficient concentration [72,73].

#### 3.1.3. Drug Metabolism

It is generally assumed that the liver is the major site of drug metabolism. Morphogenesis of the liver occurs during the 10-week GA period, the development of smooth endoplasmic reticulum begins at the 10th GW, and hepatocellular hyperplasia and hypertrophy continue until early adulthood [76]. Previous studies found that antipyrine clearance was related to age even after correction for liver weight [77,78]. Therefore, changes in drug clearance in neonates primarily depend on the maturation of transporters, intrinsic activity of liver enzymes, and regional blood flow, rather than solely on liver size [79,80]. 

Drug-metabolizing enzymes include phase I enzymes (e.g., CYP450 enzymes and non-CYP-mediated iso-enzymes) involving oxidative, reduction, and hydroxylation, and phase II enzymes (such as UGTs) involving glucuronidation, sulfation, methylation, acetylation, or glutathione conjugation reactions [81]. Because of immature drug metabolism, drug toxicity is more significant in newborns and infants than in adults [82]. The main contributors to drug clearance are CYP enzymes, and each CYP isoenzyme has its own expression and activity ontogeny profiles. Overall, the liver microsomal protein content is lower in newborns than in adults and gradually increases with age, reaching the maximum level by approximately 30 years of age [47]. The most abundant CYP450 enzyme in newborns is CYP3A7, which develops in embryonic hepatic tissue as early as a GA of 50–60 days, and its activity gradually decreases with age but is still present in many individuals until the first year of age [83,84,85]. In contrast, CYP3A4, one of the fastest-changing enzyme activities in early life, displays a mirror image pattern: the activity and expression increase in the first week of age, reaching 30% of adult levels at one month [86,87]. Owing to differences in substrate specificity and catalytic activity between CYP3A7 and CYP3A4, the individual metabolic capacity constantly changes during development and maturation. CYP2E1 can be detected in the liver as early as a GA of 93–186 days, and its expression is highly correlated with increased PNA rather than GA [88]. Non-CYP hepatic isoenzymes, including esterase, flavin-containing monooxygenases (FMOs), and alcohol or aldehyde dehydrogenases, play important roles in drug metabolism by mediating oxidative reactions. Carboxylesterases (CES) are important for insecticide detoxication [89]. In a previous study conducted by Pope et al., no significant differences were found in the expression of hepatic CES between the infants group (2–24 months) and adults (20–36 years) [90]. Moreover, hepatic CES activity was ranked as follows: 2 months < 3 months < 20 years < 24 months < 4 months < 36 years < 21 years < 8 months < 34 years < 35 years [90]. However, Yang et al. found that the mRNA and protein expression of hepatic CES was age-dependent, and its activity in adults was approximately four-fold higher than that in children and 10-fold higher than that in children and fetuses, respectively [91]. FMO1, 2, and 3 are active enzymes involved in exogenous metabolism. Similar to the age-related transition from CYP3A7 to CYP3A4 in the liver, the developmental expression of hepatic FMO1 and FMO3 showed the opposite pattern [92]. Using an optimized enzyme immunoassay, the mean aldehyde dehydrogenase content of perinatal infants was found to be approximately 10-fold lower than that in adults [93]. Phase II catalytic enzymes include glucuronosyltransferases, sulfotransferases, glutathione S-transferases, arylamine N-acetyltransferases, and methyltransferases, the activity and expression of which may be correlated with development. The expression of uridine diphosphate glucuronyltransferase in the liver is approximately 1% of adult levels during the GW of 30–40 and increases significantly to adult values by the first few weeks of life [94]. Different UGTs isoforms show different expression and activity patterns with increasing age, and all are found in the liver, early in gestation [12]. An LC-MS/MS proteomics method was used to investigate the ontogeny of six UGTs, and it was found that the protein abundances of UGT1A1, UGT1A4, UGT1A6, UGT1A9, UGT2B7, and UGT2B15 increased by approximately 8-, 55-, 35-, 33-, 8-, and 3-fold, respectively, from neonates to adults [95]. Similar to CYP450 enzymes, glucuronidation capacity maturation in neonates depends on PNA and postmenstrual age (PMA) rather than on GA [96]. Moreover, limited glucuronidated enzymes in neonates can be partly compensated by sulfate conjugation [97,98]. The expression of uridine 5′-diphospho-glucuronosyltransferase (SULTs) shows significantly different developmental patterns. For example, the protein expression of SULT1A1 did not change significantly during various developmental periods, whereas that of SULT2A1 increased during the third trimester of gestation and continued to increase after birth [99]. Information regarding the changes in the other phase II catalytic enzymes during development is presented in Table 3.

The drugs which are extracted greater than 70% by the liver are defined as high clearance drugs. Their intrinsic clearances are greater than liver blood flow. For these drugs, liver blood flow rather than enzymatic activity possesses a determinant effect on drug disposition [100]. Compared to adults, the fraction of hepatic blood flow in cardiac output is higher in children (38% vs. 24%) [101]. For neonates, hepatic function is slightly improved after birth because of an increase in neonatal hepatic blood flow related to pronounced alteration in postnatal circulatory systems [102]. 

**Table 3:** Ontogeny of phase I and phase II enzymes in human liver tissues. Fetus: 0 years, neonates: 0–1 months, infants: 1 month–2 years, children: 2–12 years, adolescents 12–16 years, adults: >16 years.

Enzymes	Measure Methods	Age-Related Changes
CYP1A1	Western blot	1. Detectable in fetal liver during 11–20 weeks, undetectable in adults [103].
	Gene expression	1. Detectable in human embryonic livers (GW 6–12), and decreases with increasing age [104,105].
CYP1A2	Western blot	1. No expression in fetal and neonatal livers, and its levels increased in infants aged 1–3 months to reach 50% of the adult value by 1 year of age [106].
	Gene expression	1. Expression was only found in adult livers [104].
CYP1B1	Gene expression	1. Detectable in fetal liver (GW 12–19) [107]. 2. Undetectable in either fetal or adult livers [108].
CYP2A6	Immunohistochemistry	1. Expression approaches adult levels by 1 year of age [109].
	Gene expression	1. Undetectable in fetal liver, increases with age [110].
CYP2B6	Immunohistochemistry	1. Approximately 10% of adult levels within the 1st year of life [109].
	Gene expression	1. Undetectable in fetal liver at GW 11–24 [110].
CYP2C8	Western blot	1. Undetected in fetal livers and matures in the first few weeks after birth, not related to GA [111].
	Gene expression	1. Low expression in fetuses, approximately 10% of the adult values [110,111].
CYP2C9	Quantitative proteomics	1. Increases linearly over age and reaches adult level in the pediatric period [92].
	Gene expression	1. Undetectable in fetal samples, comparable in pediatric population and adults [92].
CYP2C19	Quantitative proteomics	1. Expression peaked during the pediatric period (>2-fold higher compared to adult) [92].
	Western blot	1. Expression in children was 140% of that in adult liver [112].
	Gene expression	1. Undetectable in fetuses, higher expression in the pediatric population than in adults [92].
CYP2D6	Western blot	1. Undetectable in fetal livers (GW 11–13) [113]. 2. Expression in fetal liver (>GW 30) was comparable to that of newborns aged 1–7 days; increased significantly after birth and reached 50 to 75% of adult level during the neonatal period [103].
	Gene expression	1. Expression peaked in newborns and declined [113].
CYP2E1	Western blot	1. CYP2E1 was detectable in the liver as early as the second trimester; its expression in neonates was lower than that of infants 31 to 90 days less than that of older infants, children, and young adults [88]. 2. Expression increased gradually, reaching 30 to 40% of adult levels by one year and approaching adult levels by 10 years [114]. 3. Expression in fetal liver (GW 16) was about 10 to 30% of adult levels, and remained stable for up to 24 weeks [115].
	Gene expression	1. Low expression in fetal livers and increased after birth [116].
CYP2J2	Western blot	1. Expression in fetal liver (GW 13–18) was comparable to the adult level [117].
CYP3A4	Quantitative proteomics	1. Increased after birth and reached adult levels around 1 year of age [92].
	Western blot	1. Expression in fetal livers was low, and increased after birth to reach 30%–40% of adult levels [85]. 2. Expression increased with age [118]. 3. Expression in children was 60% of adult levels [112].
	Gene expression	1. Low expression in fetuses, increased during childhood, and then became comparable with adults in pediatric period [92]. 2. Expression increased rapidly after birth and reached a plateau at the first week of age [85]. 3. Only detectable after birth and was highly variable (10-fold) among adults [119]. 4. Expression exhibited a 29–fold increase after a postnatal surge [118].
CYP3A5	Quantitative proteomics	1. Comparable from fetuses to adults [92].
	Gene expression	1. Expression remained stable in fetuses, pediatrics, and adults [92]. 2. Detectable in all the fetal and 23% of adult samples [119].
CYP3A7	Quantitative proteomics	1. Very high expression in fetal samples, then decreased in the pediatrics and adults [92].
	Western blot	1. High expression in the fetal livers; its activity peaked in the first week after birth, then decreased [85].
	Gene expression	1. High expression in fetal livers and decreased with age to be undetectable in adults [92]. 2. Detectable in fetal livers at GA 50–60 days, continued to be expressed at a significant levels during the perinatal period, then decreased after first week of age until undetectable by 1 year old [85]. 3. Expression in adults was proven [119].
CYP4A1	Western blot	1. Expression in fetal livers reached 40% of the adult levels and continued to increase during the first week after birth [120].
Carboxylesterases	Western blot	1. No significant difference in expression of carboxylesterases between infants (2–24 months) and adults (20–36 years) [90]. 2. Expression was age-dependent: adult > children > fetuses [91].
	Gene expression	1. The liver expressed two major carboxylesterases: HCE1 and HCE2. Expression was age-dependent: adult > children > fetuses [91].
FMO1	Quantitative proteomics	1. High expression in fetuses, and undetectable in pediatrics and adults [92].
	Western blot	1. Highest expression in the embryo (GW 8–15) and suppressed within 3 days after birth [121].
	Gene expression	1. Higher in fetuses, decreased with age [92].
FMO3	Quantitative proteomics	1. A linear increase from the fetal period into adulthood [92].
	Western blot	1. Low expression in embryo, undetectable in the fetus, increased to be detectable by 1–2 years of age, continued to increase up to 18 years of age [121]. 2. Higher expression in children 2–8 years of age than in adults [112].
	Gene expression	1. Undetectable in fetuses, increased with age [92].
ADH1A	Western blot	1. High expression in the fetus, particularly in the first trimester, decreased in the last trimester, and finally undetected in neonates and adults [122].
ADH1B	Western blot	1. Detectable in the fetal liver at 17th GW, and dominated by week 36 [122].
ADH1C	Western blot	1. Detectable in the fetal liver at 19th GW [122].
ADH2	Gene expression	1. Detected only in fetal livers at concentrations equivalent to adults [123].
ADH3	Gene expression	1. Widely distributed in fetal tissues at concentrations equivalent to adults [123].
ADH5	Gene expression	1. Detected only in fetal livers at concentrations equivalent to adults [123].
EPHX1	Immunocytochemistry	1. Expression in fetal livers was approximately 25–50% of adult levels, and its activity was detected in fetal livers at GW 6 and increased linearly with age [124,125].
PON1	Gene expression	1. Detectable in fetal livers [126].
PON2	Gene expression	1. Detectable in fetal livers [126].
AOX	Western blot	1. Detectable in infants > 4 months old; Undetectable in infants of 13 days old and 2 months old [127].
UGT1A1	Quantitative proteomics	1. Neonatal abundances of UGT1A1 were 12.2% of adult levels whereas infant abundances (% of adult abundance) were 43; UGT1A1 is the most abundant of the UGT1As in neonates [95].
	Gene expression	1. Undetected in the fetal liver (GW 20) and stayed stable after 6 months of age [128].
UGT1A3	Gene expression	1. Undetected in the fetal liver (GW 20) and stayed stable after 6 months of age [128].
UGT1A4	Quantitative proteomics	1. Neonatal abundances of UGT1A4 were 1.8% of adult levels whereas infant abundances (% of adult abundance) were 16 [95].
	Gene expression	1. Undetected in the fetal liver (GW 20) and stayed stable after 6 months of age [128].
UGT1A6	Quantitative proteomics	1. Neonatal abundances of UGT1A6 were 2.9% of adult levels whereas infant abundances (% of adult abundance) were 15 [95].
	Gene expression	1. Undetected in the fetal liver (GW 20) and stayed stable after 6 months of age [128].
UGT1A9	Quantitative proteomics	1. Neonatal abundances of UGT1A9 were 3.0% of adult levels whereas infant abundances (% of adult abundance) were 24 [95].
	Gene expression	1. Undetected in the fetal liver (GW 20), increased with age from 6 to 24 months, reaching 70% of the adult levels [128].
UGT2B4	Gene expression	1. Undetectable in the fetal liver (GW 20), increased progressively [128].
UGT2B7	Quantitative proteomics	1. Neonatal abundances of UGT2B7 were 13.0% of adult levels whereas infant abundances (% of adult abundance) were 41 [95].
	Western blot	1. Low protein levels and activity at <1 year of age, increased progressively with age, but still less than adult levels at 17 years of age [129].
	Gene expression	1. Undetectable in the fetal liver (GW 20) and reached adult levels at 6 months of age [128].
UGT2B15	Quantitative proteomics	1. Neonatal abundances of UGT2B15 were 38.6% of adult levels whereas infant abundances (% of adult abundance) were 60 [95].
UGT2B17	Quantitative proteomics	1. Undetectable in children under 9 years, and increased by about 10-fold to reach adult levels during pubertal development [130].
SULT1A1	Western blot	1. Detectable in fetuses at GW 10 and remained stable in fetal and postnatal periods, then increased [99,131,132].
SULT1A3	Western blot	1. High expression in early fetal stage and decreased substantially in late fetal stage, then reached low levels in adults [132].
	Gene expression	1. High expression in early fetal stage and decreased substantially in late fetal stage, then reached low levels in adults [132].
SULT1C1	Gene expression	1. Low expression in fetal livers and undetectable in adult livers [133].
SULT1E1	Western blot	1. Expression peaked in the earliest gestation period [99]. 2. Higher expression in fetal livers than in adults [132].
	Gene expression	1. Detectable in fetuses at GW 10–14, slightly higher than in adults [132].
SULT2A1	Western blot	1. Low expression in fetuses at GW 25, increased to approach adult levels in neonates [99].
GSTA1/2	Starch gel electrophoresis	1. Undetectable in fetal livers before GW 30, and steadily increased to reach adult levels by PNA 1–2 years [134].
	Western blot	1. Detectable at GW 8 and rapidly increased at GW 13 [135].
GSTM	Immunohistochemistry	1. Detectable in fetal livers (GW 10–30) and then increased rapidly to adult levels by 42 weeks after birth [136]. 2. GSTM levels remained constant over pre- and postnatal period [137].
	Western blot	1. Detectable at GW 8 and slightly decreased at GW 13 [135].
GSTP1	Immunohistochemistry	1. Expression peaked in early fetal stages at GW 10–22, then decreased in the second and third trimesters, remained detectable in neonates [136].
	Western blot	1. Detectable in embryo livers at GW 8 and slightly increased at GW 13 [135].
GSTZ1	Western blot	1. Undetectable in fetal livers, increased with age until 7 years of age, remained stable between 7 and 74 years of age [138].

Abbreviations: ADH, alcohol or aldehyde dehydrogenases; AOX, aldehyde oxidase; CYP, cytochrome P450; EPHX, human cytosolic epoxide hydrolases; FMO, flavin-containing mono-oxygenase; GA, gestational age; GST, glutathione S-transferases; GW, gestational week; PNA, postnatal age; PON, paraoxonase; SULT, sulfotransferases; UGT, uridine 5′-diphospho-glucuronosyltransferase.

#### 3.1.4. Drug Excretion

In addition to metabolic elimination, most drugs and their metabolites are eliminated from the body via kidneys. The kidneys in neonates are smaller than those in adults and continue to increase in size during the juvenile and pediatric periods [139]. This increase in size is mainly dependent on the increase in tubular mass because the number of glomeruli is constant from nephrogenesis to maturation [139]. The kidneys in preterm infants are much smaller than those in term newborns, and the mean total kidney volume doubles during PMA at 28–37 weeks [140]. Generally, human nephrogenesis and vasculogenesis occur in utero and are completed at GW 36, whereas tubule maturation and growth continue during the first year after birth [141,142]. Notably, nephrogenesis in preterm neonates lasts until 40 days after birth [41]. 

Renal clearance of drugs involves three processes: glomerular filtration, tubular secretion, and active/passive tubular reabsorption [143]. Renal elimination capacity depends on renal functional maturation, a dynamic process closely correlated with morphogenesis, with significant developmental changes occurring during gestation and the first 18 months of life. A delicate balance between the renal vasoconstrictive and vasodilatory forces leads to increased renal vascular resistance after birth [144]. Therefore, the renal blood flow in neonates is low, reaching only 10% of cardiac output by the first week of life [64]. Previous research has reported that adequate renal plasma flow in premature and term infants is 20 mL/min/1.73 m^2^ and 83 mL/min/1.73 m^2^, respectively [64]. Then, effective renal plasma flow increases with age, eventually reaching the adult levels of 650 mL/min/1.73 m^2^ by 2 years of age [64]. GFR changes dramatically following the decrease in renal vascular resistance and the increase in renal blood flow in early life [4,145]. It has been reported that GFR begins immediately after birth and is approximately 40% of adult values in neonates (41 ± 15 mL/min/1.73 m^2^) [4,145]. Subsequently, GRF increases rapidly to approximately 60% of adult values (66 ± 25 mL/min/1.73 m^2^) during PNA 2–8 weeks, reaching adult levels of 100–125 mL/min/1.73 m^2^ by the age of 8–12 months [4,145,146]. In addition, the GFR in preterm infants is only half of that of newborns compared to full-term infants, as nephrogenesis is incomplete until GW 34 and initially rises slowly to reach an average level until eight years of age [12,147]. Generally, the GFR is limited to newborns and appears to be dependent on weight, GA, and PNA [148]. The active tubular secretion is also immature in neonates and is approximately 20–30% of adult levels, and then approaches the adult capacity by 7–12 months of age [4,35]. Hence, the GFR matures more rapidly than tubular secretion, leading to glomerulo-tubular imbalance in neonates [148]. Tubular reabsorption is the last renal function to develop, displays the steepest rise during 1–3 years of age, and continually increases until adolescence [148,149]. Elimination through tubular secretion and reabsorption depends on renal blood flow [35]. Tubular secretion and reabsorption are involved in active transport processes, so maturational changes in transporters must be considered for drugs with extensive renal elimination.

Furthermore, biliary excretion is the main elimination pathway for some drugs, such as polar macromolecular compounds (>300 g/mol), oral drugs highly bound to hepatic transporters, and hydrophilic drugs that extensively bind to plasma proteins for parenteral administration [150]. Bile acid concentration in the intestine is low at birth, equivalent to approximately 60% of the adult levels, and increases to approximately 80% by one year of age [151]. The bile acid pool is reduced in both premature and full-term infants, and the full-term infants experience a remarkable expansion in the size of the bile acid pool at the end of pregnancy [152,153]. The bile acid pool then continues to increase with age, reaching adult levels by two years of age but approaching adult levels by seven weeks if corrected for body surface area [154]. In addition, numerous transporters are involved in drug transport across the basal outer membrane and tubule membrane of hepatocytes, along with bile acids; therefore, developmental changes in transporters are important for drug excretion through bile.

#### 3.1.5. Transporters

Membrane transporters that are physiologically expressed throughout the body control the influx and efflux of endogenous and exogenous substances and affect the ADME process [155,156]. Investigating transporter ontogeny is challenging because of the lack of specific transporters and the unclear correlation between measured mRNA levels and actual protein expression. In general, the expression of transporters gradually increases during organogenesis [50,72,157,158]. The expression of P-glycoprotein, organic cation transporter 1 (OCT1), and organic anion-transporting polypeptide 1B3 is significantly lower at birth and increases with age. In contrast, the hepatic expression of multidrug resistance-associated protein 2 and OATP1B1 in neonates is delayed and reduced compared to that in adults until the first months of life [157,159,160]. In this study, we investigated the transporter expression in the liver, kidney, intestine, and blood–brain barrier during development (Table 4).

### 3.2. Neonatal Pathological Changes Affecting the Drug Disposition

Except for the aforementioned maturational physiological changes, including expression of metabolic enzymes and transporters, the ontogeny of the liver, kidney, and other organs or tissues, the non-maturational, pathophysiological factors, such as asphyxia, sepsis, and patent ductus arteriosus, also serve as clinically relevant variables of the drug disposition in newborns [79,172]. In general, asphyxia may induce changes in drug disposition, such as reduced or altered drug absorption, increased or unchanged volume of distribution, and decreased drug clearance [173,174]. For example, the elimination of the serum half-life and serum trough concentrations of ceftazidime were significantly increased in asphyxiated neonates, whereas the clearance and GFR of ceftazidime decreased [175]. Another study based on a popPK model found that gentamicin clearance was significantly reduced in asphyxiated neonates compared to that in non-asphyxiated neonates, and a prolonged dosing interval was necessary to achieve target trough concentrations in this subpopulation [176]. In addition to altered organ function, hemodynamics, capillary permeability, acid/base disorders, endothelial injury, and enzyme activity (e.g., CYP3A), sepsis also results in changes in PK, such as increased, decreased, or unchanged drug absorption, increased distribution of hydrophilic drugs, unchanged distribution of lipophilic drugs, and altered drug clearance in the shock state [174,177,178]. Glomerular hyperfiltration of beta-lactam drugs has been observed in adult patients with sepsis and may be associated with altered renal blood flow [179,180,181]. In neonatal sepsis, the distribution of amoxicillin significantly increases, resulting in lower exposure and a longer half-life compared to that in non-systemic disease conditions [182]. Gentamicin clearance decreased and the volume of distribution increased in neonates (<GW 36) with patent ductus arteriosus compared to those without clinically suspected patent ductus arteriosus [183]. Acute kidney injury may cause changes in hepatic blood flow and decrease CYP3A activity, further affecting drug metabolism in the neonatal liver [184]. Additionally, supporting techniques can interfere with drug PK: therapeutic hypothermia may reduce drug absorption and alter drug distribution (increased or decreased, unchanged) and elimination (decreased or unchanged); extracorporeal membrane oxygenation may induce changes in drug absorption (decreased or unchanged), distribution (increased or unchanged), and elimination (increased, decreased, or unchanged) [174].

### 3.3. Literature Search Results

**Figure 2:**
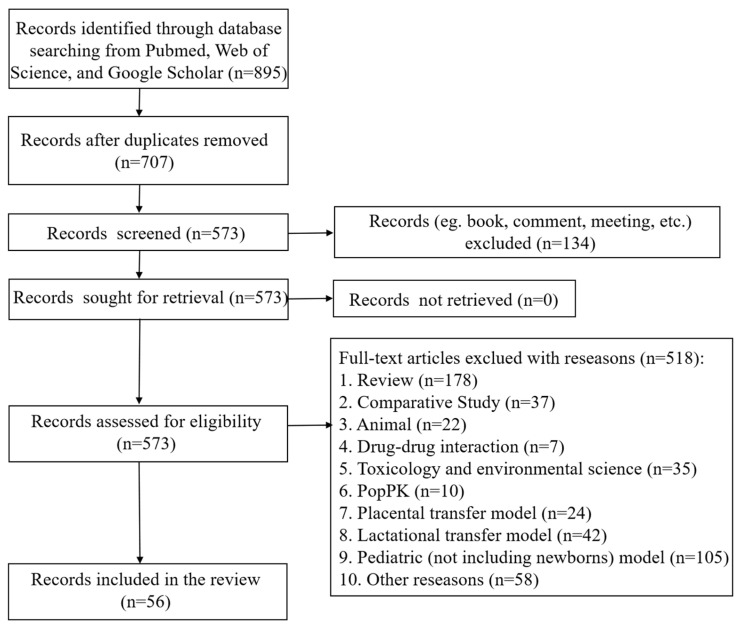
The flow chart of the literature search.

We identified a total of 895 records in the initial search. After removal of duplicates (n = 188), 707 articles were screened by reading titles and abstracts based on inclusion and exclusion criteria. We read the full text of the remaining 573 articles (Table S1) and retrieved 56 articles in the final analysis (Table 5). Full details of the search workflow are shown in Figure 2.

### 3.4. Neonatal PBPK Modeling Platform

**Figure 3:**
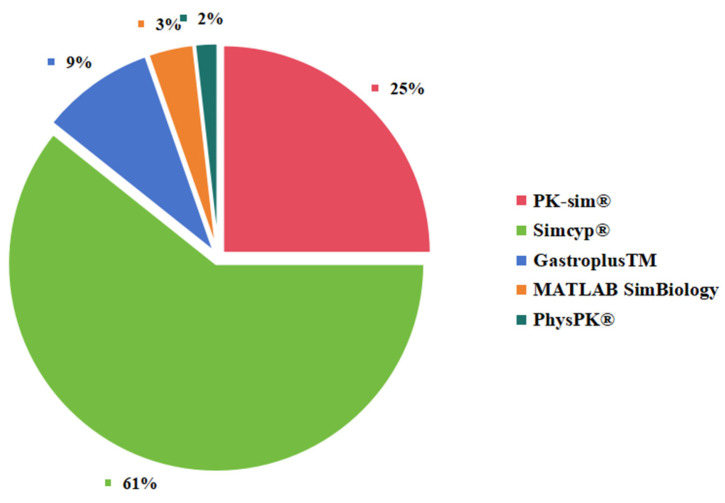
The platform of PBPK modeling used in this study.

Currently, mainstream modeling tools include Gastroplus^TM^, Simcyp^®^, PK-Sim^®^, and MATLAB Simbiology. These software programs similarly provide human multi-compartmental model structure, physiological databases, model construction tools, model evaluation tools, and visualization and reporting modules, though specific designs or technical details may be different. For example, these tools utilize age-dependent pediatric physiology data from various sources. Of these 56 articles, 34 used Simcyp^®^, 14 used PK-sim^®^, 5 used Gastroplus^TM^, 2 used MATLAB SimBiology, and 1 used PhysPK^®^ (Figure 3). As the Open Systems Pharmacology (OSP) community is a nonprofit organization, the use of PK-sim^®^ for PBPK simulation has increased in recent years. Some previous publications have presented comparisons between existing software programs [2,235,236,237,238]; we have not explored this in detail since that is not the focus of this article.

### 3.5. Application of Neonatal-PBPK Modeling for Dose Optimization/Regimens/Selection

Because of huge changes in physiological and metabolic development during the first few weeks of life, the drug disposition in neonates varies over days and dose regimens for newborns are difficult to determine in clinical practice. PBPK modeling is a potent tool used to characterize maturational changes in drug disposition and help pediatricians with treatment strategies in neonates. A total of 20 studies in this review established PBPK models and provided dose optimization, dose regimen, or dose selection for neonates. Moreover, PBPK modeling can simulate virtual bioequivalence trials to aid the evaluations of generic drug products. 

#### 3.5.1. Antiretroviral Drugs

The antiretroviral regimen is the primary approach for neonates born to HIV-infected mothers, helping reduce the mother-to-child transmission rate. Pregnant women infected with HIV are more prone to give birth to premature neonates than healthy women [239]. Neonates born prematurely to HIV-positive mothers are anatomically undeveloped and physiologically immature [201]. Therefore, exploring suitable dosing regimens of antiretroviral drugs in this population is necessary to ensure their efficacy and safety. Four antiretrovirals, nevirapine, lamivudine, raltegravir, and zidovudine, have been approved for the prevention and treatment of preterm neonates, owing to sufficient safety and PK data [240]. Wei et al. developed a whole-body PBPK model incorporating the biometric values of preterm neonates to predict zidovudine exposure in preterm neonates with different GA [201]. This simulation suggests that neonates with a lower GA display a lower capacity for drug clearance. Hence, zidovudine dosages in preterm infants may need to be adjusted for GA [201]. To explore additional potent alternative therapeutic strategies for neonates, Bunglawala et al. established a PBPK model of dolutegravir incorporated into neonatal maturation characteristics [185]. This qualified model indicated that 5 mg every 48 h for the first three weeks, followed by daily dosing during the fourth week, was suitable for preventing and treating HIV infection in newborns. 

Nafamostat, a potent serine protease inhibitor, has shown significantly higher recovery and lower mortality rates in high-risk patients with COVID-19 than that in standard care alone [241]. An IV PBPK model of nafamostat in adults was developed and scaled to the pediatric population, including newborns, to provide evidence for the use of a weight-based dosing regimen in pediatric COVID-19 patients [200]. However, insufficient PK data for nafamostat in humans have limited the predictive performance of the model. 

#### 3.5.2. Antibiotics

The empirical therapy recommended for neonates with Gram-negative infections or suspected early onset sepsis is gentamicin, an aminoglycoside antibiotic with a narrow therapeutic index, either alone or in combination with beta-lactam antibiotics [242]. The bactericidal effect of gentamicin is concentration dependent; therefore, a high maximum serum concentration (C_max_) can achieve a post-antibiotic effect [187]. However, the high trough concentration (C_min_) of gentamicin is associated with severe adverse effects such as irreversible ototoxicity and reversible nephrotoxicity [243,244,245]. As much as 79% of gentamicin is renally eliminated via glomerular filtration and undergoes tubular reabsorption [246]. Considering the immature renal function of newborns, extensive monitoring of neonates administered gentamicin is necessary. Two PBPK models have been developed to optimize the dosing regimens in neonates. One is a minimal PBPK model built for preterm and term neonates, which suggests that extended-interval dosing regimens (6 mg/kg q36h for full-term neonates and 6 mg/kg q48h for preterm neonates) possess higher efficacy and lower toxicity [187]. Another one is a PBPK-pharmacodynamic model for preterm infants, which manifests that a higher dose with an extended-dosing interval (5 mg/kg q36h) in newborns with PMA 30–34 weeks PNA 8–28 days and PMA ≥ 35 weeks PNA 0–7 days is more likely to achieve trough concentration < 1 μg/mL compared to once-daily dosing [186]. 

Meropenem, a broad-spectrum carbapenem antibiotic, has been approved by the U.S. Food and Drug Administration to treat complicated intra-abdominal infections in infants under three months of age [247]. Ganguly et al. developed a pediatric PBPK model that incorporated information on renal transporter ontogenesis and GFR maturation [33]. The predictive performance of this model was good and 90% of virtual infants achieved the target plasma concentration using a product label-recommended dosing regimen. However, this model does not consider the potential influence of disease situations and co-medications on meropenem exposure in young infants. 

Ampicillin is a beta-lactam antibiotic frequently used to treat early and late-onset neonatal sepsis [248,249]. Li and Xie developed a PBPK model of ampicillin in neonates and pregnant women during the intrapartum period and indicated that 50 mg/kg ampicillin q8h was effective and safe for newborns, and that 1 g of ampicillin was adequate for intrapartum prophylaxis [188]. 

Clindamycin is a lincosamide antibiotic used to treat anaerobic, streptococcal, and staphylococcal infections. Christoph PH et al. developed a pediatric PBPK model to optimize intravenous clindamycin dosing, and suggested a 9 mg/kg/dose for children ≤ 5 months of age, 12 mg/kg/dose for children > 5 months–6 years of age, and 10 mg/kg/dose for children 6–18 years of age, all administered every 8 h [189]. In addition, Willmann S et al. established a pediatric PBPK model covering children aged from 7 days to 18 years old for moxifloxacin and proposed an age-dependent dosing regimen: 5 mg/kg for school children (6–14 years), 7 mg/kg for pre-school children (2–6 years), and 9 mg/kg for infants and toddlers (3 months–2 years) [190]. However, this model did not support dose finding in young children (<3 months). 

Fungal-related morbidity and mortality is a major threat for most neonatal intensive care units in the world. Fluconazole is a triazole antifungal agent that is active against most of the fungal pathogens [250]. There are two PBPK models created by PK-sim^®^ to optimize the dosing regimens of fluconazole in neonates. One found that target concentration in plasma and cerebral spinal fluid was reached more quickly after using a 25 mg/kg loading dose in neonates [193]. The other one proposed a loading dose regimen for children receiving extracorporeal membrane oxygenation support in the first 24 h of therapy: 30 mg/kg for neonates, 35 mg/kg for infants and children (2 years to < 12 years), and 30 mg/kg for adolescents [194]. 

#### 3.5.3. Cardiovascular Drugs

Lisinopril is an oral long-acting synthetic peptidyl dipeptidase inhibitor of angiotensin-converting enzyme and is extensively used to treat children with hypertension [251]. This drug has high interindividual variability at all clinically used doses, ranging from 5 to 50 mg [199]. Therefore, a PBPK model was used to predict lisinopril exposure in pediatric populations across a wide age range to formulate standard treatment guidelines. The mean simulated C_max_ and area under the concentration curve (AUC; 0–120 h) of lisinopril in neonates to infants were much higher than those reported in adults, whereas time to reach C_max_ (T_max_) of lisinopril in neonates to infants was comparable to that reported for children [199]. Hence, a dose reduction is necessary if neonates and infants are administered lisinopril. 

Aminophylline is a drug comprising theophylline and ethylenediamine in a 2:1 ratio. It works for the treatment of lung diseases by inhibiting the isoenzymes phosphodiesterase III and IV [252]. It has been reported that aminophylline has benefits for the treatment of bradycardia and apnea, especially in premature infants [253]. However, aminophylline has a narrow therapeutic range of 10–20 µg/mL, which drives dosing findings in the newborn. Tummala H et al. created a PBPK model and found that a standard dosing protocol of 5 mg/kg loading dose and 2 mg/kg maintenance dose, all administrated every 8 h in preterm babies, reached the therapeutic concentration [198]. PNA had a significant influence on the clearance of aminophylline, and dosing for neonates with renal underdevelopment must be conservative.

#### 3.5.4. Antiepileptic Drugs

Carbamazepine is extensively used to prevent epileptic seizures in children and shows age-dependent changes in absorption [254]. An oral absorption model was used to improve our understanding of age-related oral absorption of carbamazepine in children using GastroPlus^TM^ [9]. This model suggested that a dose reduction was needed to obtain the desired PK results: 1.0 and 1.5 to 2.5 mg for neonates and infants to toddlers.

Valproic acid is an orally active histone deacetylase inhibitor, and is used to treat epilepsy, bipolar disorder, metabolic disease, and prevention of migraine headaches. The dosage regimens for valproic acid based on PBPK modeling are 31.25 mg for neonate, 62.5 mg for infant; 125 mg for toddler and pre-schooler; 250 mg for school age; and 375–500 mg for adolescent; all administrations are twice a day [191].

#### 3.5.5. Other Drugs

It has been reported that the clearance of propofol, which is used in general anesthesia for induction and maintenance of sedation, is sensitive to CO changes [7]. Allegaert et al. developed a PBPK model to explore the effects of CO on PK characteristics [7]. The simulation suggested that the changes in propofol clearance following reduced CO were age dependent and appeared to be lower in newborns. Furthermore, the dose of propofol was recommended to be reduced by 15% for neonates, infants, and children and 25% for adults under a low CO (40–50% reduction). 

For docetaxel, a widely used anti-mitotic chemotherapy medication, the dose recommendation based on PBPK modeling was as follows: adult dosage regimen for pediatric patients ≥ 40 kg; a weight-based regimen for pediatric patients weighing 2.5 to <40 kg (5 mg/kg loading dose on Day 1 then 2.5 mg/kg daily maintenance dose starting on Day 2) [195]. A proteomics-informed PBPK supported the FDA label dose of acetaminophen injection in neonates and infants [192]. For tadalafil, the PBPK simulation for children less than 2 years old suggested the following: children aged birth to <1 month (2 mg), children aged 1 to <6 months (3 mg), children aged 6 months to <1 year (4 mg), and children aged 1 to <2 years (6 mg) [197].

### 3.6. How Pediatricians Can Benefit from PBPK Modeling and Simulation

Although pediatricians may not be familiar with modeling software, they can leverage PBPK modeling and simulation to make clinical decisions by working with experts in the field and utilizing validated models or developing new ones tailored to specific patients. 

First, pediatricians collect relevant clinical data on the patient, including age, weight, medical history, and relevant laboratory values (e.g., liver and kidney function tests). Based on the collected data, pediatricians work with clinical pharmacologists or experts in PBPK modeling to identify the most appropriate PBPK model for the specific drug of interest. This may involve selecting a model from a library of pre-validated models or developing a new model tailored to the patient’s characteristics. The selected or developed PBPK model is then validated using internal and external data sources to ensure that it accurately predicts drug concentrations in the patient’s plasma and tissues. This validation process ensures that the model is a reliable tool for making clinical decisions. Then, using the validated PBPK model, pediatricians can simulate different dosing regimens or treatment strategies to evaluate their impact on drug concentrations and potential adverse events. These simulations take into account patient-specific factors such as age, weight, and physiological parameters derived from the PBPK model. Next, pediatricians evaluate the simulation results to determine the optimal dosing regimen or treatment strategy that balances efficacy and safety for the specific patient. This may involve comparing the predicted drug concentrations, therapeutic targets, and adverse event risks for various dosing regimens. Based on the simulation results, pediatricians develop a clinical protocol that outlines the recommended dosing regimen or treatment strategy for the patient. During the course of treatment, pediatricians monitor the patient’s clinical status and drug concentrations to assess the effectiveness of the chosen dosing regimen and identify any potential adverse events. Adjustments to the treatment plan can be made as needed based on the ongoing monitoring and re-evaluation of the PBPK model simulations.

## 4. Conclusions/Future Directions

In the field of neonatal pharmacokinetics, there is a growing need for more advanced and comprehensive PBPK models to optimize drug therapy and minimize adverse effects. Although significant progress has been made in recent years, a considerable gap remains in our understanding of the unique physiological characteristics of neonates, which hinders the development of more accurate and patient-specific PBPK models. Future studies should focus on addressing these challenges and advancing the field of neonatal PBPK modeling.

First, there is a need for a more comprehensive understanding of the ontogeny of the key organs and systems involved in drug metabolism and transport in neonates. To date, the less-studied metabolic enzymes, transporters, and population-based discrepancies in ontogeny are worthy of attention. The neonatal ontogeny of drug targets also requires continued research, as it contributes to possible differences in the exposure–response relation between neonates and older people. This knowledge will enable the development of more accurate PBPK models that account for distinct biological processes occurring during the early stages of life. To achieve this, researchers should continue to explore the molecular and cellular mechanisms underlying neonatal drug metabolism and transport and utilize animal models and in vitro systems to validate their findings.

Second, the incorporation of advanced computational methods and machine learning algorithms can significantly enhance the predictive capabilities of PBPK models. Apart from the published neonatal PBPK models, obviously more potential clinical dosing scenarios in neonates have not been studied. More examples and practices contribute to enhancing the predictive performance of PBPK modeling. Population pharmacokinetic simulation helps to construct pharmacokinetic datasets that could be used in PBPK model evaluation, which is particularly suited for neonates who cannot accept intensive blood collection. By leveraging a wealth of available data on neonatal pharmacokinetics, researchers can develop more sophisticated models that better capture the intervariability and patient-specific aspects of drug disposition in this population. This may involve the use of data mining and meta-analysis techniques to identify trends and patterns in existing data as well as the development of novel algorithms and software tools to facilitate model validation and optimization.

Third, there is a need for a more integrative approach to PBPK modeling in neo-nates, which involves the collaboration of various stakeholders, including clinicians, researchers, regulatory authorities, and the pharmaceutical industry. This multidisciplinary approach enables the efficient translation of research findings into clinical practice and ultimately leads to better therapeutic outcomes for newborns. To facilitate this process, there should be an increased focus on developing standardized guidelines and tools for PBPK model development and validation in the neonatal population as well as initiatives to promote knowledge sharing and collaboration among stakeholders.

Given the ethical and logistical challenges associated with conducting clinical re-search on neonates, it is essential to develop in silico and in vitro platforms, such as organ chips, to predict drug disposition in this population. These alternative methods can provide valuable insights into the pharmacokinetics of drugs in newborns and re-duce the need for invasive and risky clinical studies. Therefore, there should be a concerted effort to develop and validate novel in silico and in vitro models of neonatal pharmacokinetics with the ultimate goal of improving drug therapy and outcomes in this vulnerable patient population. On the other hand, to ensure the consistent development and validation of PBPK models across different regions, international collaboration and standardization efforts are vital. Consensus meetings and joint research initiatives can facilitate the exchange of knowledge and expertise, promoting the creation of a unified framework for PBPK modeling in neonates. This collaboration will help address the unique challenges and opportunities presented by the neonatal population and advance the field as a whole.

In conclusion, the future of neonatal PBPK modeling holds great promise but also presents several challenges. By focusing on these areas, researchers can advance the field and improve the lives of newborns through more effective and safer drug therapies.

## Figures and Tables

**Table 1:** List of physiological changes that affect the oral drug absorption in neonates (preterm and full-term). Fetus: 0 years, neonates: 0–1 months, infants: 1 month–2 years, children: 2–12 years, adolescents 12–16 years, adults: >16 years.

Parameters	Population	Main Performance
Gastric pH	Preterm neonates	1. Relatively higher than term infants [13].
	Neonates	1. Drops from approximately 7 to approximately 2 after birth, then rises to above 4 [42].
	Infants	1. Declines back to approximately 2 in two years [42].
	Adults	1. Approximately 1–2.
Gastric volume	Neonates	1. Decreased compared with older children and adults.
Gastric motility	Highly preterm neonates	1. Lower than full-term neonates and infants [43].
	Term neonates	1. Slower than that in older children and adults, and matures rapidly after birth [43].
	Adults	1. Biphasic emptying [51].
Intestinal pH	Neonates/infants	1. 6.6 ± 0.4 for duodenal pH; 6.6 ± 0.4 and 6.8 ± 0.7 for the pH of jejunum and ileum, respectively [52].
	Adults	1. Slightly lower than neonates [53].
Intestinal transit time	Preterm newborns	1. Approximately four-fold that of term infants [48].
	Term neonates	1. Approximately four hours proven by an in vitro model [48].
	Adults	1. Approximately four hours.
Intestinal surface area	Neonates/infants/children	1. Reduced surface-to-volume ratio compared with adults [54].
Intestinal permeability	Preterm neonates	1. The intestinal permeability in preterm neonates (GW 26–36) was higher than full-term newborns [55].
Intestinal microbiome	Preterm neonates	1. Reduced microbial diversity and increased pathogenic organism colonization vs. term neonates [56].
	Term neonates	1. Immature and matures rapidly during the first year of life [56].
Intestinal fluid composition	Neonates/infants	1. Lower bile acid and salt concentration, no secondary bile salts, and higher total protein and lipid concentrations compared to adults [57].
Digestive enzyme secretion	Preterm neonates	1. Enterokinase secretion at GW 24 is approximately 25% of the values of older infants [41]. 2. Lactase activity at GW 34 is only 30% of the levels in term neonates [58].
	Term neonates	1. Trypsin secretion reaches approximately 90% of childhood levels at term [59]. 2. Pepsin expression is not completed in neonates and matures with age [48]. 3. Pancreatic triglyceride lipase is lower in neonates than in adults [60].
Intestinal P-gp	Preterm neonates	1. Expression is lower than term infants, children, and adults [49].
	Term neonates	1. Lower than adults [49].
Intestinal CYP3A4	Neonates	1. Low expression after birth and increases from neonates to adults [46,50].

Abbreviations: CYP, cytochrome P450; GW, gestational week.

**Table 2:** List of physiological changes that affect drug distribution in neonates (preterm and full-term). Fetus: 0 years, neonates: 0–1 months, infants: 1 month–2 years, children: 2–12 years, adolescents 12–16 years, adults: >16 years.

Parameters	Population	Main Performance
Total body water (%)	Preterm neonates	As high as 90% of body weight [61,62,63].
	Term neonates	Higher than adults (80–85%) [61,62,63].
	Adults	Approximately 60–65% of body weight [61,62,63].
Total body fat (%)	Preterm neonates	3% in a premature neonate with deficient birth weight [63,64,65].
	Term neonates	12% in full-term babies [63,64,65].
Weight loss	Preterm neonates	A 10–15% weight loss at the end of first week [61].
	Term neonates	A 5–7% weight loss at the end of first week [61].
HAS levels	Preterm neonates	The mean albumin level in preterm infants (GW 23–34) is 2.36 g/dL [68].
	Term neonates	The mean albumin level in full-term neonates is 3.43 g/dL [68].
	Adults	The mean albumin level in adults is 4.0 g/dL [74].
AAG levels	Preterm newborns	Stay stable at a low level until 260 days of GA and significantly increase afterward [69].
	Term neonates	About 50% of adult level [75].

Abbreviations: AAG, alpha 1-glycoprotein; GW, gestational week, HAS, human serum albumin.

**Table 4:** The age-related changes in expression of membrane transporters in neonates. Fetus: 0 years, neonates: 0–1 months, infants: 1 month–2 years, children: 2–12 years, adolescents 12–16 years, adults: >16 years.

Transporters	Measure Methods	Expression Related to Age
**Hepatic Transporters**		
MDR1	Quantitative proteomics	1. P-gp expression was significantly lower in neonatal or infant livers and increased with age [161]. 2. Lower expression in neonates than in adults, whereas no difference between preterm and term newborns [162].
	Western blot	1. Lower expression in S9 liver fractions from children (seven days to 18 years old) than that from adults [163].
	Gene expression	1. Increase in the first years [164,165].
BCRP	Quantitative proteomics	1. Stable from neonate to adults [161,162].
	Western blot	1. Stable expression in neonates and adults [72].
	Immunohistochemistry	1. Detected in fetuses at GW 5.5 [157].
	Gene expression	1. Gene expressed in fetuses is 3-fold lower than that in adults [166]. 2. Expression in neonates is lower than children > 7 years [159].
OATP1B1	Quantitative proteomics	1. High expression in fetuses and low expression in term neonates [162]. 2. No age-dependent changes [161].
	Gene expression	1. Expression in adults > fetuses > neonates [50].
OATP1B3	Quantitative proteomics	1. Stable from fetuses to adults [162]. 2. Expression is lower in neonates than in adults and increased with age [161].
	Immunohistochemistry	1. Expressed in early childhood; increases with age [157].
	Gene expression	1. Higher in adults than in fetuses and neonates [50].
OATP2B1	Quantitative proteomics	1. Stable from neonates to adults [161,162].
	Immunohistochemistry	1. Expressed in early childhood, overexpression in neonates and young infants [157].
	Gene expression	1. Gene expression is much higher in adults than in fetuses and neonates [50].
NTCP	Quantitative proteomics	1. Stable from neonates to adults [161]. 2. Lower expression in preterm neonates than that in adults [162].
	Western blot	1. Similar expression in neonates and adults [72].
	Gene expression	1. Lower gene expression in neonates than in adults [50].
OCT1	Quantitative proteomics	1. Lower expression in term neonates than in adults [162]. 2. Lower expression in neonates than in young infants and increases to adult age [161].
	Western blot	1. Low expression in newborns, increases from birth up to 8–12 years old [167].
MRP2	Quantitative proteomics	1. Lower expression in term neonates than in adults [162]. 2. No age-dependent changes in expression [161].
	Gene expression	Lower gene expression in fetuses and neonates than that in adults [50].
MRP3	Quantitative proteomics	1. Lower expression in term neonates than in adults [162]. 2. Lower expression in infants and adolescents than that in adults [161].
	Gene expression	1. Lower gene expression in fetuses than that in adults [166].
MRP1	Quantitative proteomics	2. Lower expression in term neonates than in adults [162].
	Immunohistochemistry	1. Detectable in fetal livers [157].
MRP4	Gene expression	1. No age-dependent changes in expression [166].
MRP6	Gene expression	1. Expression increases in an age-dependent manner from neonates to adults [159].
BSEP	Quantitative proteomics	1. Lower expression in fetuses and term neonates than in adults [162]. 2. No age-dependent changes in expression [161].
	Immunohistochemistry	1. Detectable in second-trimester fetuses [168].
	Gene expression	1. Lower expression in fetuses and neonates than that in adults [159,166].
MATE1	Immunohistochemistry	1. No age-dependent changes in protein abundance [157].
	Gene expression	1. Expression increase in an age-dependent behavior [159].
GLUT1	Quantitative proteomics	1. Higher in fetal livers than in term neonates, children, and adults [162].
MCT1	Quantitative proteomics	1. Stable expression from fetuses to adults [162].
ATP1A1	Quantitative proteomics	1. Stable expression from fetuses to adults [162]. 2. Lower expression in neonates and increases with age [161].
**Renal Transporters**		
MDR1	Quantitative proteomics	1. Lowest protein abundance in neonates and reaches adult level during childhood (2–12 yr) [169].
	Immunohistochemistry	1. Detectable in the kidney by GW 5.5 [157].
	Gene expression	1. Expression in premature and/or term newborns was significantly lower than in the older age groups, no difference between preterm and term newborns [169].
BCRP	Quantitative proteomics	1. Protein abundance is similar between neonates and adults [169].
	Immunohistochemistry	1. High in newborns and reduces with age [157].
	Gene expression	1. mRNA expression is higher in term newborns than in children and adolescents [169].
MRP1	Immunohistochemistry	1. Detectable in the kidney by GW 5.5 [157].
MRP2	Gene expression	1. Expression is similar between all age groups (preterm newborn, term newborn, infants, children, adolescents, and adults [169].
MRP4	Immunohistochemistry	1. Detectable in the kidney by GW 27 [169].
	Gene expression	1. Expression is similar between all age groups (preterm newborn, term newborn, infants, children, adolescents, and adults [169].
OCT2	Quantitative proteomics	1. Protein abundance was lower in term neonates and infants than in older populations [169].
	Gene expression	1. Expression in premature and/or term newborns was significantly lower than in older age groups [169].
OAT1	Quantitative proteomics	1. Protein abundance is lowest in term newborns and infants, approaching adult levels in children or adolescents [169].
	Gene expression	1. Expression in premature and/or term newborns was significantly lower than in older age groups [169].
OAT3	Quantitative proteomics	1. Protein abundance is lowest in term newborns and infants, reaching adult levels in adolescence [169].
	Gene expression	1. Expression in premature and/or term newborns was significantly lower than in older age groups [169].
URAT1	Quantitative proteomics	1. Protein abundance is lower in term newborns and infants, reaching adult levels during childhood [169].
	Gene expression	1. mRNA expression in infants and children is higher in term neonates and adults [169].
MATE1	Quantitative proteomics	1. No age-dependent changes in expression [169].
	Gene expression	1. No age-dependent changes in expression [169].
**Transporters in the blood–** **brain barrier**		
MDR1	Immunohistochemistry	1. Low expression at birth (approximately 30% to 50% of adults), increases with postnatal maturation, reaching adult levels at around 3–6 months [170].
**Intestinal transporters**		
MDR1	Quantitative proteomics	1. Similar levels in preterm newborns, full-term neonates, and adults [161,162].
	Immunohistochemistry	1. Similar between children of different ages and adults [157].
	Gene expression	1. Detectable at GW 14-22 [171]. 2. Expression in duodenal and jejunal was stable in children from 1 month to adulthood [49]. 3. Expression in neonatal and infant intestines is similar to that in adult intestines [50].
MRP2	Immunohistochemistry	1. Similar between children of different ages and adults [157].
	Gene expression	1. Stable in expression from neonates to adults [50].
OATP2B1	Quantitative proteomics	1. Similar levels in preterm newborns, full-term neonates, and adults [162].
	Immunohistochemistry	1. Higher expression in neonates and young infants than in adults [157].
	Gene expression	2. Higher expression in neonates than in adults [50].

Abbreviations: ATP1A1, ATPase Na+/K+ transporting subunit alpha 1; BCRP, breast cancer resistance protein; BSEP, bile salt export pump; GLUT, glucose transporter; GW, gestational week; MATE, multidrug and toxin extrusion; MCT, Monocarboxylate transporter; MDR, multidrug resistance gene; MRP, multidrug resistance-associated protein; NTCP, sodium/taurocholate cotransporting polypeptide; OAT, organic anion transporter; OATP, organic anion transporting polypeptide; OCT, organic cation transporter; P-gp, P-glycoprotein; TM_50_, age at which half of the adult value is reached; URAT, uric acid transporter 1.

**Table 5:** Neonatal PBPK modeling studies.

Study Types/Ref.	Drugs	Platform	Main Outcomes
**Dose Optimization/Regimens/Selection**			
[185]	Dolutegravir	MATLAB SimBiology	1. Recommended regimen for neonates: Day 1–20 = 5 mg every 48 h (q48h); Day 21–28 = 5 mg every 24 h (q24h). 2. If the mother has taken dolutegravir 2–24 h before delivery, the first dose for the newborn may be delayed until 24–48 h after birth.
[186]	Gentamicin	Simcyp^®^	1. This model suggested that a higher dose with an extended-dosing interval (5 mg/kg q36h) was beneficial for neonates with PMA 30–34 weeks PNA 8–28 days and PMA ≥ 35 weeks PNA 0–7 days.
[187]	Gentamicin	PhysPK^®^	1. Extended-interval dosing regimens (6 mg/kg q36h and 6 mg/kg q48h for term and preterm neonates) were recommended because of higher efficacy and lower toxicity.
[188]	Ampicillin	Simcyp^®^	1. Recommended dosing regimens: 50 mg/kg q8h for neonates; 1 g ampicillin for a duration ≤ 4 h before delivery for intrapartum prophylaxis.
[189]	Clindamycin	PK-Sim^®^	1. The optimal dosing supported by this PBPK model was 9 mg/kg/dose for children ≤ 5 months of age, 12 mg/kg/dose for children > 5 months–6 years of age, and 10 mg/kg/dose for children 6–18 years of age, all administered every 8 h.
[190]	Moxifloxacin	PK-Sim^®^	1. Initial dosing regimen based on PBPK model: 5 mg/kg for school children (6–14 years), 7 mg/kg for pre-school children (2–6 years), and 9 mg/kg for infants and toddlers (3 months–2 years). 2. The popPK model suggested that an alternative dosing regimen (200 mg q12h i.v.) can be developed for children aged 12–18 years.
[191]	Valproic acid	Simcyp^®^	1. Dosage recommendation (bid): 31.25 mg for neonate, 62.5 mg for infants; 125 mg for toddler and pre-schooler; 250 mg for school age; 375–500 mg for adolescent.
[192]	Acetaminophen	GastroPlus^TM^	1. A proteomics-informed PBPK supported the FDA label dose of acetaminophen injection in neonates and infants.
[193]	Fluconazole	PK-sim^®^	1. Target drug concentration in plasma and CSF was reached more quickly after using a 25 mg/kg loading dose in neonates.
[194]	Fluconazole	PK-sim^®^	1. Loading dose recommendations for children on ECMO in the first 24 h of therapy: 30 mg/kg for neonates, 35 mg/kg for infants and children (2 years to <12 years), and 30 mg/kg for adolescents.
[195]	Docetaxel	Simcyp^®^	1. The PBPK simulation suggested that the revised dose of docetaxel for a child > 1.5 years old was higher than the adult dose, while this was the same for children aged 1 to 1.5 years.
[7]	Propofol	Simcyp^®^	1. The change in propofol clearance following CO reductions increased with age. 2. If CO was reduced by 40–50%, the dose of propofol should be reduced by 15% for newborns, infants, and children, and 25% for adolescents and adults.
[196]	Remdesivir	Simcyp^®^	1. Dose recommendation: adult dosage regimen for pediatric patients ≥ 40 kg; a weight-based regimen for pediatric patients weighing 2.5 to <40 kg (5 mg/kg loading dose on Day 1 then 2.5 mg/kg daily maintenance dose starting on Day 2).
[197]	Tadalafil	Simcyp^®^	1. A PBPK model was developed to explore tadalafil doses for children less than 2 years old: children aged birth to <1 month (2 mg), children aged 1 to <6 months (3 mg), children aged 6 months to <1 year (4 mg), and children aged 1 to <2 years (6 mg).
[198]	Aminophylline	PK-sim^®^	1. PNA had a significant influence on clearance of the drug. 2. Dosing for neonates with renal underdevelopment should be conservative.
[33]	Meropenem	PK-Sim^®^	1. This PBPK model supported the meropenem dosing regimens recommended in the product label for infants < 3 months of age with complicated intraabdominal.
[199]	Lisinopril	PK-Sim^®^	1. 1.0 and 1.5 to 2.5 mg for neonates to infants and infants to toddlers; 5 and 10 mg for adolescents; 20 mg for adults.
[200]	Nafamostat	Simcyp^®^	1. A whole-body PBPK model of nafamostat in adults was built and scaled to children including newborns, providing evidence for using a weight-based dosing regimen in pediatric COVID-19 patients.
[201]	Zidovudine	PK-sim^®^	1. The preterm newborns of lesser GA have less capacity for drug clearance. 2. Zidovudine dosages in preterm infants may need to be adjusted for GA.
[9]	Carbamazepine	GastroPlus^TM^	1. Dose reduction was recommended: 1.0 and 1.5 to 2.5 mg for neonates and infants to toddlers.
**Special application of neonatal PBPK modeling**			
[202]	Oseltamivir	GastroPlus^TM^	1. The generic drugs with 10% slower dissolution profile than the reference drugs could maintain BE in adults, while the dissolution boundary for pediatrics is restricted (6% slower for adolescents, 4% slower for 0–2-month neonates) to maintain BE.
**Neonatal PBPK model establishment and simulation**			
[203]	Oseltamivir	Simcyp^®^	1. The protein abundance data of hepatic CESs were imported into a pediatric PBPK model and the concentration of oseltamivir in infants (0–1 year of age) was successfully predicted.
[204]	Oseltamivir	GastroPlus^TM^	1. The exposure of intravenous oseltamivir in neonates was 3-fold higher than those observed with the same oral doses.
[205]	Morphine	Simcyp^®^	1. The clearance of morphine increased from preterm to term to post-term neonates. 2. OCT1 genotype influences morphine clearance in term and post-term neonates.
[206]	Morphine	Simcyp^®^	1. Morphine clearance in neonates was more sensitive to developmental changes in UGT2B7 activity.
[207]	Morphine	Simcyp^®^	1. The variation in BBB expression of P-gp transporter was not responsible for differences in brain exposure of the drug. 2. The PBPK-PD modeling suggested that neonates were more sensitive to morphine than adults and older children.
[208]	Methadone	Simcyp^®^	1. Changes in CYP2B6 and CYP3A4 activity, AGP, and MPPGL affected the drug clearance in neonates. 2. The effect of cardiac output on drug disposition was negligible.
[209]	Propofol	Simcyp^®^	1. A PBPK model combining in vitro and in vivo data made a good prediction on clearance and concentration–time profiles of propofol across a broad age span from preterm to adults.
[210]	Buprenorphine	PK-Sim^®^	1. A whole-body parent-metabolite PBPK model of buprenorphine for an adult and two pediatric populations (children aged 4.6–7.5 years and preterm neonates with PMA 27–34 weeks) was successfully developed and displayed an excellent predictive model performance.
[211]	Buprenorphine	Simcyp^®^	1. The PK variability of buprenorphine in neonates was influenced by the extent of biliary clearance, oral mucosa absorption, and CYP3A4 abundance.
[212]	Dihydrocodeine	Simcyp^®^	1. A full PBPK model of dihydrocodeine for healthy adults was established and scaled to pediatric patients with varied ages (from 1 month old to 14 years old).
[213]	Fentanyl	PK-Sim^®^	1. A whole-body parent-metabolite PBPK model of fentanyl for adults was built and successfully scaled to several pediatric subpopulations (from preterm neonates to up to 3-year-old children).
[214]	Sirolimus	Simcyp^®^	1. The maturation pattern of sirolimus clearance in pediatric patients was successfully investigated through a novel PBPK model.
[215]	Actinomycin D	Simcyp^®^	1. A PBPK model of actinomycin D in infants and younger children was established with perfect predictive performance.
[216]	Gentamicin	PK-Sim^®^	1. The simulation found a good correlation between plasma and saliva exposures, supporting saliva concentration as an alternative for TDM of gentamicin in premature infants.
[217]	Midazolam	PK-sim^®^	1. The predicted midazolam disposition in preterm neonates was comparable to the observed data. 2. The MRT in neonatal brain was higher than the MRT in plasma.
[218]	Acetaminophen	Simcyp^®^	1. A pediatric PBPK model integrating physiological changes and enzyme ontogeny successfully described the PK profiles from birth to adulthood (0–17 years).
[219]	Fluconazole	Simcyp^®^	1. A model-based bridging approach (from juvenile mice/adults/in vitro–in silico data to neonates) successfully predicted fluconazole PK profiles in neonates.
[220]	Apixaban	Simcyp^®^	1. Apixaban levels reduced with weight-normalized clearance in infants younger than 1 year old.
[221]	Bumetanide	Simcyp^®^	1. This model suggested that the brain concentration of bumetanide in neonates enrolled in NEMO clinical trial was lower than therapeutic concentration.
[222]	Acetaminophen	Simcyp^®^	1. The validated model found that a reduced CO by up to 30% did not affect drug disposition in preterm neonates.
[223]	HSK3486	Simcyp^®^	1. No change in the systemic exposure of HSK386 in neonates compared to that in adults.
[224]	Hydrocortisone	Simcyp^®^	1. This hydrocortisone PBPK model successfully predicted PK parameters of both immediate- and modified-release hydrocortisone formulations in adults and pediatrics.
[101]	Theophylline and midazolam	MATLAB SimBiology	1. Total clearance of these two drugs was very low in neonates and increased by 5 years old, then decreased in adults.
[225]	Ganciclovir and valganciclovir	GastroPlus^TM^	1. The drug exposure in neonates was slightly under-predicted through this modeling.
[226]	Pantoprazole and esomeprazole	Simcyp^®^	1. The interplay of CYP maturation and inhibition in neonates might be age-dependent.
[227]	Sildenafil and phenytoin	Simcyp^®^	1. The PK of both sildenafil and phenytoin were predicted better at the end of a prolonged study using the time-varying compared to fixed PBPK models.
[228]	Cimetidine, ciprofloxacin, metformin, tenofovir, and zidovudine	Simcyp^®^	1. Predictions in neonates and early infants (up to 14 weeks PNA) were reasonable.
[229]	Recombinant human erythropoietin, infliximab, etanercept, basiliximab, anakinra, and enfuvirtide	Simcyp^®^	1. A PBPK model incorporating age-dependent physiology changes was developed to predict PK profiles of different therapeutic proteins in the pediatric population, including full-term neonates.
[8]	Theophylline, paracetamol, and ketoconazole	Simcyp^®^	1. A pediatric absorption model integrating the available information on pediatric gastrointestinal physiology and its ontogeny was developed to predict oral drug absorption.
[230]	Paracetamol, alfentanil, morphine, theophylline, and levofloxacin	PK-Sim^®^	1. The plasma concentration in neonates was greater than that in the adults, and the concentration in older children was less than that in the adults. 2. No age-dependent bias for term neonates to 18 years of age when examining volumes of distribution and t_1/2_.
[231]	Meropenem, ceftazidime, azithromycin, propofol, midazolam, lorazepam, and caffeine	Simcyp^®^	1. The predictive performance of this PBPK model appeared to decrease in the (pre)term neonatal population.
[21]	Midazolam, caffeine, carbamazepine, cisapride, theophylline, diclofenac, omeprazole, S-warfarin, phenytoin, gentamicin, and vancomycin	Simcyp^®^	1. The PBPK modeling using Simcyp^®^ software was more accurate than that of simple allometry scaling, especially in small children (<2 years old)
[232]	Linezolid and emtricitabine	Simcyp^®^	1. A PBPK model incorporating renal maturation ontogeny successfully predicted the PK of linezolid and emtricitabine in the pediatric population, including neonates.
[233]	Alfentanil, midazolam, caffeine, ibuprofen, gentamicin, and vancomycin	Simcyp^®^	1. All PK parameter predictions of these six drugs for preterm neonates were within twofold error criteria.
[234]	Amikacin, ciprofloxacin, copanlisib, gadovist and magnevist, levonorgestrel, moxifloxacin, regorafenib, riociguat, and rivaroxaban	PK-Sim^®^	1. The pediatric PBPK models successfully predict the PK profiles of 10 small-molecule compounds in children with different age.

Abbreviations: AGP, α1-acid glycoprotein; BBB, blood–brain barrier; CESs, carboxylesterases; BE, bioequivalence; bid, twice a day; CO, cardiac output; CSF, cerebral spinal fluid; CYP, cytochrome p450; ECMO, extracorporeal membrane oxygenation; GA, gestational age; i.v., intravenous; MPPGL, the microsomal protein per gram of liver; MRT, mean residence time; OCT, organic cation transporter; PBPK, physiologically based pharmacokinetic; PD, pharmacodynamic; P-gp, P-glycoprotein; PK, pharmacokinetic; PMA, postmenstrual age; PNA, postnatal age; TDM, therapeutic drug monitoring; t_1/2_, half-life; UGT, uridine 5′-diphospho-glucuronosyltransferase.

## Data Availability

No new data were created or analyzed in this study. Data sharing is not applicable to this study.

## Supplementary Materials

The following supporting information can be downloaded at https://www.mdpi.com/article/10.3390/pharmaceutics15122765/s1, Table S1: List of neonatal PBPK modeling dataset.

## References

[1] Laughon M.M., Avant D., Tripathi N., Hornik C.P., Cohen-Wolkowiez M., Clark R.H., Smith P.B., Rodriguez W. (2014). Drug labeling and exposure in neonates. JAMA Pediatr..

[2] Zhou X., Dun J., Chen X., Xiang B., Dang Y., Cao D. (2023). Predicting the correct dose in children: Role of computational Pediatric Physiological-based pharmacokinetics modeling tools. CPT Pharmacomet. Syst. Pharmacol..

[3] Cella M., Knibbe C., Danhof M., Della Pasqua O. (2010). What is the right dose for children?. Br. J. Clin. Pharmacol..

[4] Kearns G.L., Abdel-Rahman S.M., Alander S.W., Blowey D.L., Leeder J.S., Kauffman R.E. (2003). Developmental pharmacology--drug disposition, action, and therapy in infants and children. N. Engl. J. Med..

[5] Kaneria N.S., Tuleu C., Ernest T. (2022). Opportunities for enteral drug delivery for neonates, infants, and toddlers: A critical exploration. Expert Opin. Drug Deliv..

[6] Smits A., Annaert P., Allegaert K. (2013). Drug disposition and clinical practice in neonates: Cross talk between developmental physiology and pharmacology. Int. J. Pharm..

[7] Allegaert K., Abbasi M.Y., Michelet R., Olafuyi O. (2022). The Impact of Low Cardiac Output on Propofol Pharmacokinetics across Age Groups-An Investigation Using Physiologically Based Pharmacokinetic Modelling. Pharmaceutics.

[8] Johnson T.N., Bonner J.J., Tucker G.T., Turner D.B., Jamei M. (2018). Development and applications of a physiologically-based model of paediatric oral drug absorption. Eur. J. Pharm. Sci..

[9] Kohlmann P., Stillhart C., Kuentz M., Parrott N. (2017). Investigating Oral Absorption of Carbamazepine in Pediatric Populations. AAPS J..

[10] Lu H., Rosenbaum S. (2014). Developmental pharmacokinetics in pediatric populations. J. Pediatr. Pharmacol. Ther..

[11] Badee J., Qiu N., Parrott N., Collier A.C., Schmidt S., Fowler S. (2019). Optimization of Experimental Conditions of Automated Glucuronidation Assays in Human Liver Microsomes Using a Cocktail Approach and Ultra-High Performance Liquid Chromatography-Tandem Mass Spectrometry. Drug Metab. Dispos..

[12] Badee J., Fowler S., de Wildt S.N., Collier A.C., Schmidt S., Parrott N. (2019). The Ontogeny of UDP-glucuronosyltransferase Enzymes, Recommendations for Future Profiling Studies and Application Through Physiologically Based Pharmacokinetic Modelling. Clin. Pharmacokinet..

[13] Wang K., Jiang K., Wei X., Li Y., Wang T., Song Y. (2021). Physiologically Based Pharmacokinetic Models Are Effective Support for Pediatric Drug Development. AAPS PharmSciTech.

[14] Van den Anker J., Reed M.D., Allegaert K., Kearns G.L. (2018). Developmental Changes in Pharmacokinetics and Pharmacodynamics. J. Clin. Pharmacol..

[15] Mahmood I. (2016). Prediction of Drug Clearance in Premature and Mature Neonates, Infants, and Children ≤ 2 Years of Age: A Comparison of the Predictive Performance of 4 Allometric Models. J. Clin. Pharmacol..

[16] Mahmood I. (2015). Prediction of drug clearance in children: A review of different methodologies. Expert Opin. Drug Metab. Toxicol..

[17] Mahmood I., Staschen C.M., Goteti K. (2014). Prediction of drug clearance in children: An evaluation of the predictive performance of several models. AAPS J..

[18] Mahmood I. (2014). Dosing in children: A critical review of the pharmacokinetic allometric scaling and modelling approaches in paediatric drug development and clinical settings. Clin. Pharmacokinet..

[19] Edginton A.N., Shah B., Sevestre M., Momper J.D. (2013). The integration of allometry and virtual populations to predict clearance and clearance variability in pediatric populations over the age of 6 years. Clin. Pharmacokinet..

[20] Mansoor N., Ahmad T., Alam Khan R., Sharib S.M., Mahmood I. (2019). Prediction of Clearance and Dose of Midazolam in Preterm and Term Neonates: A Comparative Study Between Allometric Scaling and Physiologically Based Pharmacokinetic Modeling. Am. J. Ther..

[21] Johnson T.N., Rostami-Hodjegan A., Tucker G.T. (2006). Prediction of the clearance of eleven drugs and associated variability in neonates, infants and children. Clin. Pharmacokinet..

[22] Leong R., Vieira M.L., Zhao P., Mulugeta Y., Lee C.S., Huang S.M., Burckart G.J. (2012). Regulatory experience with physiologically based pharmacokinetic modeling for pediatric drug trials. Clin. Pharmacol. Ther..

[23] Johnson T.N. (2008). The problems in scaling adult drug doses to children. Arch. Dis. Child..

[24] Manolis E., Osman T.E., Herold R., Koenig F., Tomasi P., Vamvakas S., Saint Raymond A. (2011). Role of modeling and simulation in pediatric investigation plans. Paediatr. Anaesth..

[25] Jones H.M., Gardner I.B., Collard W.T., Stanley P.J., Oxley P., Hosea N.A., Plowchalk D., Gernhardt S., Lin J., Dickins M. (2011). Simulation of human intravenous and oral pharmacokinetics of 21 diverse compounds using physiologically based pharmacokinetic modelling. Clin. Pharmacokinet..

[26] Jones H.M., Parrott N., Jorga K., Lave T. (2006). A novel strategy for physiologically based predictions of human pharmacokinetics. Clin. Pharmacokinet..

[27] Bjorkman S. (2003). Reduction and lumping of physiologically based pharmacokinetic models: Prediction of the disposition of fentanyl and pethidine in humans by successively simplified models. J. Pharmacokinet. Pharmacodyn..

[28] Edginton A.N., Theil F.P., Schmitt W., Willmann S. (2008). Whole body physiologically-based pharmacokinetic models: Their use in clinical drug development. Expert Opin. Drug Metab. Toxicol..

[29] Nestorov I. (2007). Whole-body physiologically based pharmacokinetic models. Expert Opin. Drug Metab. Toxicol..

[30] Edginton A.N., Schmitt W., Voith B., Willmann S. (2006). A mechanistic approach for the scaling of clearance in children. Clin. Pharmacokinet..

[31] Page M.J., McKenzie J.E., Bossuyt P.M., Boutron I., Hoffmann T.C., Mulrow C.D., Shamseer L., Tetzlaff J.M., Akl E.A., Brennan S.E. (2021). The PRISMA 2020 statement: An updated guideline for reporting systematic reviews. Rev. Esp. Cardiol..

[32] Maharaj A.R., Edginton A.N. (2014). Physiologically based pharmacokinetic modeling and simulation in pediatric drug development. CPT Pharmacomet. Syst. Pharmacol..

[33] Ganguly S., Edginton A.N., Gerhart J.G., Cohen-Wolkowiez M., Greenberg R.G., Gonzalez D., Best Pharmaceuticals for Children Act-Pediatric Trials Network Steering Committee (2021). Physiologically Based Pharmacokinetic Modeling of Meropenem in Preterm and Term Infants. Clin. Pharmacokinet..

[34] Carry M.R., Ringel S.P., Starcevich J.M. (1986). Distribution of capillaries in normal and diseased human skeletal muscle. Muscle Nerve.

[35] Tayman C., Rayyan M., Allegaert K. (2011). Neonatal pharmacology: Extensive interindividual variability despite limited size. J. Pediatr. Pharmacol. Ther..

[36] Afsar F.S. (2010). Physiological skin conditions of preterm and term neonates. Clin. Exp. Dermatol..

[37] Lin Y.C., Sussman H.H., Benitz W.E. (1997). Plasma concentrations after rectal administration of acetaminophen in preterm neonates. Paediatr. Anaesth..

[38] Burri P.H. (1984). Fetal and postnatal development of the lung. Annu. Rev. Physiol..

[39] El-Gendy N., Kaviratna A., Berkland C., Dhar P. (2013). Delivery and performance of surfactant replacement therapies to treat pulmonary disorders. Ther. Deliv..

[40] Jamei M., Turner D., Yang J., Neuhoff S., Polak S., Rostami-Hodjegan A., Tucker G. (2009). Population-based mechanistic prediction of oral drug absorption. AAPS J..

[41] Butranova O.I., Ushkalova E.A., Zyryanov S.K., Chenkurov M.S. (2023). Developmental Pharmacokinetics of Antibiotics Used in Neonatal ICU: Focus on Preterm Infants. Biomedicines.

[42] Avery G.B., Randolph J.G., Weaver T. (1966). Gastric acidity in the first day of life. Pediatrics.

[43] Gan J., Bornhorst G.M., Henrick B.M., German J.B. (2018). Protein Digestion of Baby Foods: Study Approaches and Implications for Infant Health. Mol. Nutr. Food Res..

[44] Indrio F., Neu J., Pettoello-Mantovani M., Marchese F., Martini S., Salatto A., Aceti A. (2022). Development of the Gastrointestinal Tract in Newborns as a Challenge for an Appropriate Nutrition: A Narrative Review. Nutrients.

[45] Lebenthal E., Lee P.C., Heitlinger L.A. (1983). Impact of development of the gastrointestinal tract on infant feeding. J. Pediatr..

[46] Verscheijden L.F.M., Koenderink J.B., Johnson T.N., de Wildt S.N., Russel F.G.M. (2020). Physiologically-based pharmacokinetic models for children: Starting to reach maturation?. Pharmacol. Ther..

[47] Allegaert K., Mian P., van den Anker J.N. (2017). Developmental Pharmacokinetics in Neonates: Maturational Changes and Beyond. Curr. Pharm. Des..

[48] Bourlieu C., Menard O., Bouzerzour K., Mandalari G., Macierzanka A., Mackie A.R., Dupont D. (2014). Specificity of infant digestive conditions: Some clues for developing relevant in vitro models. Crit. Rev. Food Sci. Nutr..

[49] van Kalken C.K., Giaccone G., van der Valk P., Kuiper C.M., Hadisaputro M.M., Bosma S.A., Scheper R.J., Meijer C.J., Pinedo H.M. (1992). Multidrug resistance gene (P-glycoprotein) expression in the human fetus. Am. J. Pathol..

[50] Mooij M.G., Schwarz U.I., de Koning B.A., Leeder J.S., Gaedigk R., Samsom J.N., Spaans E., van Goudoever J.B., Tibboel D., Kim R.B. (2014). Ontogeny of human hepatic and intestinal transporter gene expression during childhood: Age matters. Drug Metab. Dispos..

[51] Linakis M.W., Roberts J.K., Lala A.C., Spigarelli M.G., Medlicott N.J., Reith D.M., Ward R.M., Sherwin C.M. (2016). Challenges Associated with Route of Administration in Neonatal Drug Delivery. Clin. Pharmacokinet..

[52] Barbero G.J., Runge G., Fischer D., Crawford M.N., Torres F.E., Gyorgy P. (1952). Investigations on the bacterial flora, pH, and sugar content in the intestinal tract of infants. J. Pediatr..

[53] Fredrikzon B., Olivecrona T. (1978). Decrease of lipase and esterase activities in intestinal contents of newborn infants during test meals. Pediatr. Res..

[54] Nicolas J.M., Bouzom F., Hugues C., Ungell A.L. (2017). Oral drug absorption in pediatrics: The intestinal wall, its developmental changes and current tools for predictions. Biopharm. Drug Dispos..

[55] van Elburg R.M., Fetter W.P., Bunkers C.M., Heymans H.S. (2003). Intestinal permeability in relation to birth weight and gestational and postnatal age. Arch. Dis. Child. Fetal Neonatal Ed..

[56] Scholtens P.A., Oozeer R., Martin R., Amor K.B., Knol J. (2012). The early settlers: Intestinal microbiology in early life. Annu. Rev. Food Sci. Technol..

[57] de Waal T., Brouwers J., Mols R., Hoffman I., Rayyan M., Augustijns P. (2023). Characterization of neonatal and infant enterostomy fluids. Int. J. Pharm..

[58] Lebenthal A., Lebenthal E. (1999). The ontogeny of the small intestinal epithelium. JPEN J. Parenter. Enteral Nutr..

[59] Lebenthal E., Lee P.C. (1980). Development of functional responses in human exocrine pancreas. Pediatrics.

[60] Carriere F., Barrowman J.A., Verger R., Laugier R. (1993). Secretion and contribution to lipolysis of gastric and pancreatic lipases during a test meal in humans. Gastroenterology.

[61] Lindower J.B. (2017). Water balance in the fetus and neonate. Semin. Fetal Neonatal Med..

[62] Ward R.M., Benjamin D., Barrett J.S., Allegaert K., Portman R., Davis J.M., Turner M.A. (2017). Safety, dosing, and pharmaceutical quality for studies that evaluate medicinal products (including biological products) in neonates. Pediatr. Res..

[63] Young A., Brown L.K., Ennis S., Beattie R.M., Johnson M.J. (2021). Total body water in full-term and preterm newborns: Systematic review and meta-analysis. Arch. Dis. Child. Fetal Neonatal Ed..

[64] Allegaert K., Cossey V., van den Anker J.N. (2015). Dosing Guidelines of Aminoglycosides in Neonates: A Balance Between Physiology and Feasibility. Curr. Pharm. Des..

[65] Allegaert K., van den Anker J.N. (2017). Perinatal and neonatal use of paracetamol for pain relief. Semin. Fetal Neonatal Med..

[66] Schmidt S., Gonzalez D., Derendorf H. (2010). Significance of protein binding in pharmacokinetics and pharmacodynamics. J. Pharm. Sci..

[67] Weaving G., Batstone G.F., Jones R.G. (2016). Age and sex variation in serum albumin concentration: An observational study. Ann. Clin. Biochem..

[68] Chen C.B., Hammo B., Barry J., Radhakrishnan K. (2021). Overview of Albumin Physiology and its Role in Pediatric Diseases. Curr. Gastroenterol. Rep..

[69] Anell-Olofsson M., Ahmadi S., Lonnqvist P.A., Eksborg S., von Horn H., Bartocci M. (2018). Plasma concentrations of alpha-1-acid glycoprotein in preterm and term newborns: Influence of mode of delivery and implications for plasma protein binding of local anaesthetics. Br. J. Anaesth..

[70] Nau H., Luck W., Kuhnz W. (1984). Decreased serum protein binding of diazepam and its major metabolite in the neonate during the first postnatal week relate to increased free fatty acid levels. Br. J. Clin. Pharmacol..

[71] Notarianni L.J. (1990). Plasma protein binding of drugs in pregnancy and in neonates. Clin. Pharmacokinet..

[72] Yanni S.B., Smith P.B., Benjamin D.K., Augustijns P.F., Thakker D.R., Annaert P.P. (2011). Higher clearance of micafungin in neonates compared with adults: Role of age-dependent micafungin serum binding. Biopharm. Drug Dispos..

[73] Pokorna P., Wildschut E.D., Vobruba V., van den Anker J.N., Tibboel D. (2015). The Impact of Hypothermia on the Pharmacokinetics of Drugs Used in Neonates and Young Infants. Curr. Pharm. Des..

[74] Sethi P.K., White C.A., Cummings B.S., Hines R.N., Muralidhara S., Bruckner J.V. (2016). Ontogeny of plasma proteins, albumin and binding of diazepam, cyclosporine, and deltamethrin. Pediatr. Res..

[75] McNamara P.J., Alcorn J. (2002). Protein binding predictions in infants. AAPS PharmSci.

[76] Alcorn J., McNamara P.J. (2002). Ontogeny of hepatic and renal systemic clearance pathways in infants: Part I. Clin. Pharmacokinet..

[77] Sharer J.E., Wrighton S.A. (1996). Identification of the human hepatic cytochromes P450 involved in the in vitro oxidation of antipyrine. Drug Metab. Dispos..

[78] Engel G., Hofmann U., Heidemann H., Cosme J., Eichelbaum M. (1996). Antipyrine as a probe for human oxidative drug metabolism: Identification of the cytochrome P450 enzymes catalyzing 4-hydroxyantipyrine, 3-hydroxymethylantipyrine, and norantipyrine formation. Clin. Pharmacol. Ther..

[79] Allegaert K., Simons S.H.P., Tibboel D., Krekels E.H., Knibbe C.A., van den Anker J.N. (2017). Non-maturational covariates for dynamic systems pharmacology models in neonates, infants, and children: Filling the gaps beyond developmental pharmacology. Eur. J. Pharm. Sci..

[80] Hines R.N. (2007). Ontogeny of human hepatic cytochromes P450. J. Biochem. Mol. Toxicol..

[81] Mahmood I., Ahmad T., Mansoor N., Sharib S.M. (2017). Prediction of Clearance in Neonates and Infants (≤3 Months of Age) for Drugs That Are Glucuronidated: A Comparative Study Between Allometric Scaling and Physiologically Based Pharmacokinetic Modeling. J. Clin. Pharmacol..

[82] Johnson T.N., Rostami-Hodjegan A. (2011). Resurgence in the use of physiologically based pharmacokinetic models in pediatric clinical pharmacology: Parallel shift in incorporating the knowledge of biological elements and increased applicability to drug development and clinical practice. Paediatr. Anaesth..

[83] Chen H., Brzezinski M.R., Fantel A.G., Juchau M.R. (1999). Catalysis of drug oxidation during embryogenesis in human hepatic tissues using imipramine as a model substrate. Drug Metab. Dispos..

[84] Hakkola J., Raunio H., Purkunen R., Saarikoski S., Vahakangas K., Pelkonen O., Edwards R.J., Boobis A.R., Pasanen M. (2001). Cytochrome P450 3A expression in the human fetal liver: Evidence that CYP3A5 is expressed in only a limited number of fetal livers. Biol. Neonate.

[85] Lacroix D., Sonnier M., Moncion A., Cheron G., Cresteil T. (1997). Expression of CYP3A in the human liver--evidence that the shift between CYP3A7 and CYP3A4 occurs immediately after birth. Eur. J. Biochem..

[86] de Wildt S.N., Kearns G.L., Leeder J.S., van den Anker J.N. (1999). Cytochrome P450 3A: Ontogeny and drug disposition. Clin. Pharmacokinet..

[87] Ince I., Knibbe C.A., Danhof M., de Wildt S.N. (2013). Developmental changes in the expression and function of cytochrome P450 3A isoforms: Evidence from in vitro and in vivo investigations. Clin. Pharmacokinet..

[88] Johnsrud E.K., Koukouritaki S.B., Divakaran K., Brunengraber L.L., Hines R.N., McCarver D.G. (2003). Human hepatic CYP2E1 expression during development. J. Pharmacol. Exp. Ther..

[89] Maxwell D.M. (1992). The specificity of carboxylesterase protection against the toxicity of organophosphorus compounds. Toxicol. Appl. Pharmacol..

[90] Pope C.N., Karanth S., Liu J., Yan B. (2005). Comparative carboxylesterase activities in infant and adult liver and their in vitro sensitivity to chlorpyrifos oxon. Regul. Toxicol. Pharmacol..

[91] Yang D., Pearce R.E., Wang X., Gaedigk R., Wan Y.J., Yan B. (2009). Human carboxylesterases HCE1 and HCE2: Ontogenic expression, inter-individual variability and differential hydrolysis of oseltamivir, aspirin, deltamethrin and permethrin. Biochem. Pharmacol..

[92] Zane N.R., Chen Y., Wang M.Z., Thakker D.R. (2018). Cytochrome P450 and flavin-containing monooxygenase families: Age-dependent differences in expression and functional activity. Pediatr. Res..

[93] Tran M.N., Wu A.H., Hill D.W. (2007). Alcohol dehydrogenase and catalase content in perinatal infant and adult livers: Potential influence on neonatal alcohol metabolism. Toxicol. Lett..

[94] Watchko J.F., Lin Z. (2010). Exploring the genetic architecture of neonatal hyperbilirubinemia. Semin. Fetal Neonatal Med..

[95] Bhatt D.K., Mehrotra A., Gaedigk A., Chapa R., Basit A., Zhang H., Choudhari P., Boberg M., Pearce R.E., Gaedigk R. (2019). Age- and Genotype-Dependent Variability in the Protein Abundance and Activity of Six Major Uridine Diphosphate-Glucuronosyltransferases in Human Liver. Clin. Pharmacol. Ther..

[96] Allegaert K., Vanhaesebrouck S., Verbesselt R., van den Anker J.N. (2009). In vivo glucuronidation activity of drugs in neonates: Extensive interindividual variability despite their young age. Ther. Drug Monit..

[97] Levy G., Khanna N.N., Soda D.M., Tsuzuki O., Stern L. (1975). Pharmacokinetics of acetaminophen in the human neonate: Formation of acetaminophen glucuronide and sulfate in relation to plasma bilirubin concentration and D-glucaric acid excretion. Pediatrics.

[98] McRorie T.I., Lynn A.M., Nespeca M.K., Opheim K.E., Slattery J.T. (1992). The maturation of morphine clearance and metabolism. Am. J. Dis. Child..

[99] Duanmu Z., Weckle A., Koukouritaki S.B., Hines R.N., Falany J.L., Falany C.N., Kocarek T.A., Runge-Morris M. (2006). Developmental expression of aryl, estrogen, and hydroxysteroid sulfotransferases in pre- and postnatal human liver. J. Pharmacol. Exp. Ther..

[100] Nies A.S., Shand D.G., Wilkinson G.R. (1976). Altered hepatic blood flow and drug disposition. Clin. Pharmacokinet..

[101] Bjorkman S. (2005). Prediction of drug disposition in infants and children by means of physiologically based pharmacokinetic (PBPK) modelling: Theophylline and midazolam as model drugs. Br. J. Clin. Pharmacol..

[102] Gow P.J., Ghabrial H., Smallwood R.A., Morgan D.J., Ching M.S. (2001). Neonatal hepatic drug elimination. Pharmacol. Toxicol..

[103] Shimada T., Yamazaki H., Mimura M., Wakamiya N., Ueng Y.F., Guengerich F.P., Inui Y. (1996). Characterization of microsomal cytochrome P450 enzymes involved in the oxidation of xenobiotic chemicals in human fetal liver and adult lungs. Drug Metab. Dispos..

[104] Yang H.Y., Namkung M.J., Juchau M.R. (1995). Expression of functional cytochrome P4501A1 in human embryonic hepatic tissues during organogenesis. Biochem. Pharmacol..

[105] Omiecinski C.J., Redlich C.A., Costa P. (1990). Induction and developmental expression of cytochrome P450IA1 messenger RNA in rat and human tissues: Detection by the polymerase chain reaction. Cancer Res..

[106] Sonnier M., Cresteil T. (1998). Delayed ontogenesis of CYP1A2 in the human liver. Eur. J. Biochem..

[107] Shimada T., Yamazaki H., Mimura M., Inui Y., Guengerich F.P. (1994). Interindividual variations in human liver cytochrome P-450 enzymes involved in the oxidation of drugs, carcinogens and toxic chemicals: Studies with liver microsomes of 30 Japanese and 30 Caucasians. J. Pharmacol. Exp. Ther..

[108] Hakkola J., Pasanen M., Pelkonen O., Hukkanen J., Evisalmi S., Anttila S., Rane A., Mantyla M., Purkunen R., Saarikoski S. (1997). Expression of CYP1B1 in human adult and fetal tissues and differential inducibility of CYP1B1 and CYP1A1 by Ah receptor ligands in human placenta and cultured cells. Carcinogenesis.

[109] Tateishi T., Nakura H., Asoh M., Watanabe M., Tanaka M., Kumai T., Takashima S., Imaoka S., Funae Y., Yabusaki Y. (1997). A comparison of hepatic cytochrome P450 protein expression between infancy and postinfancy. Life Sci..

[110] Hakkola J., Pasanen M., Purkunen R., Saarikoski S., Pelkonen O., Maenpaa J., Rane A., Raunio H. (1994). Expression of xenobiotic-metabolizing cytochrome P450 forms in human adult and fetal liver. Biochem. Pharmacol..

[111] Treluyer J.M., Gueret G., Cheron G., Sonnier M., Cresteil T. (1997). Developmental expression of CYP2C and CYP2C-dependent activities in the human liver: In-vivo/in-vitro correlation and inducibility. Pharmacogenetics.

[112] Yanni S.B., Annaert P.P., Augustijns P., Ibrahim J.G., Benjamin D.K., Thakker D.R. (2010). In vitro hepatic metabolism explains higher clearance of voriconazole in children versus adults: Role of CYP2C19 and flavin-containing monooxygenase 3. Drug Metab. Dispos..

[113] Treluyer J.M., Jacqz-Aigrain E., Alvarez F., Cresteil T. (1991). Expression of CYP2D6 in developing human liver. Eur. J. Biochem..

[114] Vieira I., Sonnier M., Cresteil T. (1996). Developmental expression of CYP2E1 in the human liver. Hypermethylation control of gene expression during the neonatal period. Eur. J. Biochem..

[115] Carpenter S.P., Lasker J.M., Raucy J.L. (1996). Expression, induction, and catalytic activity of the ethanol-inducible cytochrome P450 (CYP2E1) in human fetal liver and hepatocytes. Mol. Pharmacol..

[116] Boutelet-Bochan H., Huang Y., Juchau M.R. (1997). Expression of CYP2E1 during embryogenesis and fetogenesis in human cephalic tissues: Implications for the fetal alcohol syndrome. Biochem. Biophys. Res. Commun..

[117] Gu J., Su T., Chen Y., Zhang Q.Y., Ding X. (2000). Expression of biotransformation enzymes in human fetal olfactory mucosa: Potential roles in developmental toxicity. Toxicol. Appl. Pharmacol..

[118] Chen Y.T., Trzoss L., Yang D., Yan B. (2015). Ontogenic expression of human carboxylesterase-2 and cytochrome P450 3A4 in liver and duodenum: Postnatal surge and organ-dependent regulation. Toxicology.

[119] Schuetz J.D., Beach D.L., Guzelian P.S. (1994). Selective expression of cytochrome P450 CYP3A mRNAs in embryonic and adult human liver. Pharmacogenetics.

[120] Cresteil T. (1998). Onset of xenobiotic metabolism in children: Toxicological implications. Food Addit. Contam..

[121] Koukouritaki S.B., Simpson P., Yeung C.K., Rettie A.E., Hines R.N. (2002). Human hepatic flavin-containing monooxygenases 1 (FMO1) and 3 (FMO3) developmental expression. Pediatr. Res..

[122] Smith M., Hopkinson D.A., Harris H. (1971). Developmental changes and polymorphism in human alcohol dehydrogenase. Ann. Hum. Genet..

[123] Estonius M., Svensson S., Hoog J.O. (1996). Alcohol dehydrogenase in human tissues: Localisation of transcripts coding for five classes of the enzyme. FEBS Lett..

[124] Omiecinski C.J., Aicher L., Swenson L. (1994). Developmental expression of human microsomal epoxide hydrolase. J. Pharmacol. Exp. Ther..

[125] Cresteil T., Beaune P., Kremers P., Celier C., Guengerich F.P., Leroux J.P. (1985). Immunoquantification of epoxide hydrolase and cytochrome P-450 isozymes in fetal and adult human liver microsomes. Eur. J. Biochem..

[126] Mackness B., Beltran-Debon R., Aragones G., Joven J., Camps J., Mackness M. (2010). Human tissue distribution of paraoxonases 1 and 2 mRNA. IUBMB Life.

[127] Tayama Y., Sugihara K., Sanoh S., Miyake K., Kitamura S., Ohta S. (2012). Developmental changes of aldehyde oxidase activity and protein expression in human liver cytosol. Drug Metab. Pharmacokinet..

[128] Strassburg C.P., Strassburg A., Kneip S., Barut A., Tukey R.H., Rodeck B., Manns M.P. (2002). Developmental aspects of human hepatic drug glucuronidation in young children and adults. Gut.

[129] Zaya M.J., Hines R.N., Stevens J.C. (2006). Epirubicin glucuronidation and UGT2B7 developmental expression. Drug Metab. Dispos..

[130] Bhatt D.K., Basit A., Zhang H., Gaedigk A., Lee S.B., Claw K.G., Mehrotra A., Chaudhry A.S., Pearce R.E., Gaedigk R. (2018). Hepatic Abundance and Activity of Androgen- and Drug-Metabolizing Enzyme UGT2B17 Are Associated with Genotype, Age, and Sex. Drug Metab. Dispos..

[131] Richard K., Hume R., Kaptein E., Stanley E.L., Visser T.J., Coughtrie M.W. (2001). Sulfation of thyroid hormone and dopamine during human development: Ontogeny of phenol sulfotransferases and arylsulfatase in liver, lung, and brain. J. Clin. Endocrinol. Metab..

[132] Stanley E.L., Hume R., Coughtrie M.W. (2005). Expression profiling of human fetal cytosolic sulfotransferases involved in steroid and thyroid hormone metabolism and in detoxification. Mol. Cell. Endocrinol..

[133] Her C., Kaur G.P., Athwal R.S., Weinshilboum R.M. (1997). Human sulfotransferase SULT1C1: cDNA cloning, tissue-specific expression, and chromosomal localization. Genomics.

[134] Strange R.C., Davis B.A., Faulder C.G., Cotton W., Bain A.D., Hopkinson D.A., Hume R. (1985). The human glutathione S-transferases: Developmental aspects of the GST1, GST2, and GST3 loci. Biochem. Genet..

[135] Raijmakers M.T., Steegers E.A., Peters W.H. (2001). Glutathione S-transferases and thiol concentrations in embryonic and early fetal tissues. Hum. Reprod..

[136] Strange R.C., Howie A.F., Hume R., Matharoo B., Bell J., Hiley C., Jones P., Beckett G.J. (1989). The development expression of alpha-, mu- and pi-class glutathione S-transferases in human liver. Biochim. Biophys. Acta.

[137] Beckett G.J., Howie A.F., Hume R., Matharoo B., Hiley C., Jones P., Strange R.C. (1990). Human glutathione S-transferases: Radioimmunoassay studies on the expression of alpha-, mu- and pi-class isoenzymes in developing lung and kidney. Biochim. Biophys. Acta.

[138] Li W., Gu Y., James M.O., Hines R.N., Simpson P., Langaee T., Stacpoole P.W. (2012). Prenatal and postnatal expression of glutathione transferase zeta 1 in human liver and the roles of haplotype and subject age in determining activity with dichloroacetate. Drug Metab. Dispos..

[139] Frazier K.S. (2017). Species Differences in Renal Development and Associated Developmental Nephrotoxicity. Birth Defects Res..

[140] Kandasamy Y., Rudd D., Smith R., Lumbers E.R., Wright I.M. (2018). Extra uterine development of preterm kidneys. Pediatr. Nephrol..

[141] Solhaug M.J., Bolger P.M., Jose P.A. (2004). The developing kidney and environmental toxins. Pediatrics.

[142] Bueters R., Bael A., Gasthuys E., Chen C., Schreuder M.F., Frazier K.S. (2020). Ontogeny and Cross-species Comparison of Pathways Involved in Drug Absorption, Distribution, Metabolism, and Excretion in Neonates (Review): Kidney. Drug Metab. Dispos..

[143] Mahmood I. (2022). Prediction of total and renal clearance of renally secreted drugs in neonates and infants (≤3 months of age). J. Clin. Transl. Res..

[144] Filler G., Bhayana V., Schott C., Diaz-Gonzalez de Ferris M.E. (2021). How should we assess renal function in neonates and infants?. Acta Paediatr..

[145] Loebstein R., Koren G. (1998). Clinical pharmacology and therapeutic drug monitoring in neonates and children. Pediatr. Rev..

[146] Rhodin M.M., Anderson B.J., Peters A.M., Coulthard M.G., Wilkins B., Cole M., Chatelut E., Grubb A., Veal G.J., Keir M.J. (2009). Human renal function maturation: A quantitative description using weight and postmenstrual age. Pediatr. Nephrol..

[147] Iacobelli S., Loprieno S., Bonsante F., Latorre G., Esposito L., Gouyon J.B. (2007). Renal function in early childhood in very low birthweight infants. Am. J. Perinatol..

[148] Zhang Y., Mehta N., Muhari-Stark E., Burckart G.J., van den Anker J., Wang J. (2019). Pediatric Renal Ontogeny and Applications in Drug Development. J. Clin. Pharmacol..

[149] van den Anker J., Allegaert K. (2021). Considerations for Drug Dosing in Premature Infants. J. Clin. Pharmacol..

[150] Thabit A.K. (2020). Antibiotics in the Biliary Tract: A Review of the Pharmacokinetics and Clinical Outcomes of Antibiotics Penetrating the Bile and Gallbladder Wall. Pharmacotherapy.

[151] Balistreri W.F. (1983). Immaturity of hepatic excretory function and the ontogeny of bile acid metabolism. J. Pediatr. Gastroenterol. Nutr..

[152] Subbiah M.T., Hassan A.S. (1982). Development of bile acid biogenesis and its significance in cholesterol homeostasis. Adv. Lipid Res..

[153] Grijalva J., Vakili K. (2013). Neonatal liver physiology. Semin. Pediatr. Surg..

[154] Heubi J.E., Balistreri W.F., Suchy F.J. (1982). Bile salt metabolism in the first year of life. J. Lab. Clin. Med..

[155] Ho R.H., Kim R.B. (2005). Transporters and drug therapy: Implications for drug disposition and disease. Clin. Pharmacol. Ther..

[156] Shitara Y., Sato H., Sugiyama Y. (2005). Evaluation of drug-drug interaction in the hepatobiliary and renal transport of drugs. Annu. Rev. Pharmacol. Toxicol..

[157] Konieczna A., Erdosova B., Lichnovska R., Jandl M., Cizkova K., Ehrmann J. (2011). Differential expression of ABC transporters (MDR1, MRP1, BCRP) in developing human embryos. J. Mol. Histol..

[158] Kumar V., Prasad B., Patilea G., Gupta A., Salphati L., Evers R., Hop C.E., Unadkat J.D. (2015). Quantitative transporter proteomics by liquid chromatography with tandem mass spectrometry: Addressing methodologic issues of plasma membrane isolation and expression-activity relationship. Drug Metab. Dispos..

[159] Klaassen C.D., Aleksunes L.M. (2010). Xenobiotic, bile acid, and cholesterol transporters: Function and regulation. Pharmacol. Rev..

[160] Deo A.K., Prasad B., Balogh L., Lai Y., Unadkat J.D. (2012). Interindividual variability in hepatic expression of the multidrug resistance-associated protein 2 (MRP2/ABCC2): Quantification by liquid chromatography/tandem mass spectrometry. Drug Metab. Dispos..

[161] Prasad B., Gaedigk A., Vrana M., Gaedigk R., Leeder J.S., Salphati L., Chu X., Xiao G., Hop C., Evers R. (2016). Ontogeny of Hepatic Drug Transporters as Quantified by LC-MS/MS Proteomics. Clin. Pharmacol. Ther..

[162] van Groen B.D., van de Steeg E., Mooij M.G., van Lipzig M.M.H., de Koning B.A.E., Verdijk R.M., Wortelboer H.M., Gaedigk R., Bi C., Leeder J.S. (2018). Proteomics of human liver membrane transporters: A focus on fetuses and newborn infants. Eur. J. Pharm. Sci..

[163] Abanda N.N., Riches Z., Collier A.C. (2017). Lobular Distribution and Variability in Hepatic ATP Binding Cassette Protein B1 (ABCB1, P-gp): Ontogenetic Differences and Potential for Toxicity. Pharmaceutics.

[164] Mooij M.G., van de Steeg E., van Rosmalen J., Windster J.D., de Koning B.A., Vaes W.H., van Groen B.D., Tibboel D., Wortelboer H.M., de Wildt S.N. (2016). Proteomic Analysis of the Developmental Trajectory of Human Hepatic Membrane Transporter Proteins in the First Three Months of Life. Drug Metab. Dispos..

[165] Burgess K.S., Philips S., Benson E.A., Desta Z., Gaedigk A., Gaedigk R., Segar M.W., Liu Y., Skaar T.C. (2015). Age-Related Changes in MicroRNA Expression and Pharmacogenes in Human Liver. Clin. Pharmacol. Ther..

[166] Sharma S., Ellis E.C., Gramignoli R., Dorko K., Tahan V., Hansel M., Mattison D.R., Caritis S.N., Hines R.N., Venkataramanan R. (2013). Hepatobiliary disposition of 17-OHPC and taurocholate in fetal human hepatocytes: A comparison with adult human hepatocytes. Drug Metab. Dispos..

[167] Hahn D., Emoto C., Vinks A.A., Fukuda T. (2017). Developmental Changes in Hepatic Organic Cation Transporter OCT1 Protein Expression from Neonates to Children. Drug Metab. Dispos..

[168] Chen H.L., Chen H.L., Liu Y.J., Feng C.H., Wu C.Y., Shyu M.K., Yuan R.H., Chang M.H. (2005). Developmental expression of canalicular transporter genes in human liver. J. Hepatol..

[169] Cheung K.W.K., van Groen B.D., Spaans E., van Borselen M.D., de Bruijn A., Simons-Oosterhuis Y., Tibboel D., Samsom J.N., Verdijk R.M., Smeets B. (2019). A Comprehensive Analysis of Ontogeny of Renal Drug Transporters: mRNA Analyses, Quantitative Proteomics, and Localization. Clin. Pharmacol. Ther..

[170] Lam J., Baello S., Iqbal M., Kelly L.E., Shannon P.T., Chitayat D., Matthews S.G., Koren G. (2015). The ontogeny of P-glycoprotein in the developing human blood-brain barrier: Implication for opioid toxicity in neonates. Pediatr. Res..

[171] Miki Y., Suzuki T., Tazawa C., Blumberg B., Sasano H. (2005). Steroid and xenobiotic receptor (SXR), cytochrome P450 3A4 and multidrug resistance gene 1 in human adult and fetal tissues. Mol. Cell. Endocrinol..

[172] Bodenham A., Shelly M.P., Park G.R. (1988). The altered pharmacokinetics and pharmacodynamics of drugs commonly used in critically ill patients. Clin. Pharmacokinet..

[173] Cristea S., Smits A., Kulo A., Knibbe C.A.J., van Weissenbruch M., Krekels E.H.J., Allegaert K. (2017). Amikacin Pharmacokinetics To Optimize Dosing in Neonates with Perinatal Asphyxia Treated with Hypothermia. Antimicrob. Agents Chemother..

[174] Raffaeli G., Pokorna P., Allegaert K., Mosca F., Cavallaro G., Wildschut E.D., Tibboel D. (2019). Drug Disposition and Pharmacotherapy in Neonatal ECMO: From Fragmented Data to Integrated Knowledge. Front. Pediatr..

[175] Van Den Anker J.N., Van Der Heijden B.J., Hop W.C., Schoemaker R.C., Broerse H.M., Neijens H.J., De Groot R. (1995). The effect of asphyxia on the pharmacokinetics of ceftazidime in the term newborn. Pediatr. Res..

[176] Frymoyer A., Meng L., Bonifacio S.L., Verotta D., Guglielmo B.J. (2013). Gentamicin pharmacokinetics and dosing in neonates with hypoxic ischemic encephalopathy receiving hypothermia. Pharmacotherapy.

[177] Samardzic J., Allegaert K., Wilbaux M., Pfister M., van den Anker J.N. (2016). Quantitative clinical pharmacology practice for optimal use of antibiotics during the neonatal period. Expert Opin. Drug Metab. Toxicol..

[178] Vet N.J., de Hoog M., Tibboel D., de Wildt S.N. (2011). The effect of inflammation on drug metabolism: A focus on pediatrics. Drug Discov. Today.

[179] Chytra I., Stepan M., Benes J., Pelnar P., Zidkova A., Bergerova T., Pradl R., Kasal E. (2012). Clinical and microbiological efficacy of continuous versus intermittent application of meropenem in critically ill patients: A randomized open-label controlled trial. Crit. Care.

[180] Udy A.A., Varghese J.M., Altukroni M., Briscoe S., McWhinney B.C., Ungerer J.P., Lipman J., Roberts J.A. (2012). Subtherapeutic initial beta-lactam concentrations in select critically ill patients: Association between augmented renal clearance and low trough drug concentrations. Chest.

[181] Prowle J.R., Molan M.P., Hornsey E., Bellomo R. (2012). Measurement of renal blood flow by phase-contrast magnetic resonance imaging during septic acute kidney injury: A pilot investigation. Crit. Care Med..

[182] D’Agate S., Musuamba F.T., Della Pasqua O. (2020). Dose Rationale for Amoxicillin in Neonatal Sepsis When Referral Is Not Possible. Front. Pharmacol..

[183] Williams B.S., Ransom J.L., Gal P., Carlos R.Q., Smith M., Schall S.A. (1997). Gentamicin pharmacokinetics in neonates with patent ductus arteriosus. Crit. Care Med..

[184] Lewis S.J., Mueller B.A. (2016). Antibiotic Dosing in Patients with Acute Kidney Injury: “Enough But Not Too Much”. J. Intensive Care Med..

[185] Bunglawala F., Rajoli R.K.R., Mirochnick M., Owen A., Siccardi M. (2020). Prediction of dolutegravir pharmacokinetics and dose optimization in neonates via physiologically based pharmacokinetic (PBPK) modelling. J. Antimicrob. Chemother..

[186] Neeli H., Hanna N., Abduljalil K., Cusumano J., Taft D.R. (2021). Application of Physiologically Based Pharmacokinetic-Pharmacodynamic Modeling in Preterm Neonates to Guide Gentamicin Dosing Decisions and Predict Antibacterial Effect. J. Clin. Pharmacol..

[187] Zazo H., Lagarejos E., Prado-Velasco M., Sanchez-Herrero S., Serna J., Rueda-Ferreiro A., Martin-Suarez A., Calvo M.V., Perez-Blanco J.S., Lanao J.M. (2022). Physiologically-based pharmacokinetic modelling and dosing evaluation of gentamicin in neonates using PhysPK. Front. Pharmacol..

[188] Li S., Xie F. (2023). Foetal and neonatal exposure prediction and dosing evaluation for ampicillin using a physiologically-based pharmacokinetic modelling approach. Br. J. Clin. Pharmacol..

[189] Hornik C.P., Wu H., Edginton A.N., Watt K., Cohen-Wolkowiez M., Gonzalez D. (2017). Development of a Pediatric Physiologically-Based Pharmacokinetic Model of Clindamycin Using Opportunistic Pharmacokinetic Data. Clin. Pharmacokinet..

[190] Willmann S., Frei M., Sutter G., Coboeken K., Wendl T., Eissing T., Lippert J., Stass H. (2019). Application of Physiologically-Based and Population Pharmacokinetic Modeling for Dose Finding and Confirmation During the Pediatric Development of Moxifloxacin. CPT Pharmacomet. Syst. Pharmacol..

[191] Conner T.M., Nikolian V.C., Georgoff P.E., Pai M.P., Alam H.B., Sun D., Reed R.C., Zhang T. (2018). Physiologically based pharmacokinetic modeling of disposition and drug-drug interactions for valproic acid and divalproex. Eur. J. Pharm. Sci..

[192] Ladumor M.K., Bhatt D.K., Gaedigk A., Sharma S., Thakur A., Pearce R.E., Leeder J.S., Bolger M.B., Singh S., Prasad B. (2019). Ontogeny of Hepatic Sulfotransferases and Prediction of Age-Dependent Fractional Contribution of Sulfation in Acetaminophen Metabolism. Drug Metab. Dispos..

[193] Gerhart J.G., Watt K.M., Edginton A., Wade K.C., Salerno S.N., Benjamin D.K., Smith P.B., Hornik C.P., Cohen-Wolkowiez M., Duara S. (2019). Physiologically-Based Pharmacokinetic Modeling of Fluconazole Using Plasma and Cerebrospinal Fluid Samples From Preterm and Term Infants. CPT Pharmacomet. Syst. Pharmacol..

[194] Watt K.M., Cohen-Wolkowiez M., Barrett J.S., Sevestre M., Zhao P., Brouwer K.L.R., Edginton A.N. (2018). Physiologically Based Pharmacokinetic Approach to Determine Dosing on Extracorporeal Life Support: Fluconazole in Children on ECMO. CPT Pharmacomet. Syst. Pharmacol..

[195] Thai H.T., Mazuir F., Cartot-Cotton S., Veyrat-Follet C. (2015). Optimizing pharmacokinetic bridging studies in paediatric oncology using physiologically-based pharmacokinetic modelling: Application to docetaxel. Br. J. Clin. Pharmacol..

[196] Lutz J.D., Mathias A., German P., Pikora C., Reddy S., Kirby B.J. (2021). Physiologically-Based Pharmacokinetic Modeling of Remdesivir and Its Metabolites to Support Dose Selection for the Treatment of Pediatric Patients with COVID-19. Clin. Pharmacol. Ther..

[197] Rehmel J., Ferguson-Sells L., Morse B.L., Li B., Dickinson G.L. (2022). Physiologically based pharmacokinetic modeling of tadalafil to inform pediatric dose selection in children with pulmonary arterial hypertension. CPT Pharmacomet. Syst. Pharmacol..

[198] Tummala H.P., Balusu R., Thotakura S., Pasnoor A.K., Raju A.P., Lal S.M., Lewis L.E., Mallayasamy S. (2022). Development of Physiologically Based Pharmacokinetic Model and Assessment of the Impact of Renal Underdevelopment in Preterm Infants on the Pharmacokinetics of Aminophylline. J. Pharmacol. Pharmacother..

[199] Rashid M., Sarfraz M., Arafat M., Hussain A., Abbas N., Sadiq M.W., Rasool M.F., Bukhari N.I. (2020). Prediction of lisinopril pediatric dose from the reference adult dose by employing a physiologically based pharmacokinetic model. BMC Pharmacol. Toxicol..

[200] Cho Y.S., Shin J.G. (2022). Physiologically-based pharmacokinetic modeling of nafamostat to support dose selection for treatment of pediatric patients with COVID-19. Transl. Clin. Pharmacol..

[201] Wei L., Mansoor N., Khan R.A., Czejka M., Ahmad T., Ahmed M., Ali M., Yang D.H. (2019). WB-PBPK approach in predicting zidovudine pharmacokinetics in preterm neonates. Biopharm. Drug Dispos..

[202] Miao L., Mousa Y.M., Zhao L., Raines K., Seo P., Wu F. (2020). Using a Physiologically Based Pharmacokinetic Absorption Model to Establish Dissolution Bioequivalence Safe Space for Oseltamivir in Adult and Pediatric Populations. AAPS J..

[203] Boberg M., Vrana M., Mehrotra A., Pearce R.E., Gaedigk A., Bhatt D.K., Leeder J.S., Prasad B. (2017). Age-Dependent Absolute Abundance of Hepatic Carboxylesterases (CES1 and CES2) by LC-MS/MS Proteomics: Application to PBPK Modeling of Oseltamivir In Vivo Pharmacokinetics in Infants. Drug Metab. Dispos..

[204] Parrott N., Davies B., Hoffmann G., Koerner A., Lave T., Prinssen E., Theogaraj E., Singer T. (2011). Development of a physiologically based model for oseltamivir and simulation of pharmacokinetics in neonates and infants. Clin. Pharmacokinet..

[205] Hahn D., Emoto C., Euteneuer J.C., Mizuno T., Vinks A.A., Fukuda T. (2019). Influence of OCT1 Ontogeny and Genetic Variation on Morphine Disposition in Critically Ill Neonates: Lessons From PBPK Modeling and Clinical Study. Clin. Pharmacol. Ther..

[206] Emoto C., Johnson T.N., Neuhoff S., Hahn D., Vinks A.A., Fukuda T. (2018). PBPK Model of Morphine Incorporating Developmental Changes in Hepatic OCT1 and UGT2B7 Proteins to Explain the Variability in Clearances in Neonates and Small Infants. CPT Pharmacomet. Syst. Pharmacol..

[207] Verscheijden L.F.M., Litjens C.H.C., Koenderink J.B., Mathijssen R.H.J., Verbeek M.M., de Wildt S.N., Russel F.G.M. (2021). Physiologically based pharmacokinetic/pharmacodynamic model for the prediction of morphine brain disposition and analgesia in adults and children. PLoS Comput. Biol..

[208] McPhail B.T., Emoto C., Fukuda T., Butler D., Wiles J.R., Akinbi H., Vinks A.A. (2020). Utilizing Pediatric Physiologically Based Pharmacokinetic Models to Examine Factors That Contribute to Methadone Pharmacokinetic Variability in Neonatal Abstinence Syndrome Patients. J. Clin. Pharmacol..

[209] Michelet R., Van Bocxlaer J., Allegaert K., Vermeulen A. (2018). The use of PBPK modeling across the pediatric age range using propofol as a case. J. Pharmacokinet. Pharmacodyn..

[210] Kovar L., Schrapel C., Selzer D., Kohl Y., Bals R., Schwab M., Lehr T. (2020). Physiologically-Based Pharmacokinetic (PBPK) Modeling of Buprenorphine in Adults, Children and Preterm Neonates. Pharmaceutics.

[211] van Hoogdalem M.W., Johnson T.N., McPhail B.T., Kamatkar S., Wexelblatt S.L., Ward L.P., Christians U., Akinbi H.T., Vinks A.A., Mizuno T. (2022). Physiologically-Based Pharmacokinetic Modeling to Investigate the Effect of Maturation on Buprenorphine Pharmacokinetics in Newborns with Neonatal Opioid withdrawal Syndrome. Clin. Pharmacol. Ther..

[212] Ota M., Shimizu M., Kamiya Y., Emoto C., Fukuda T., Yamazaki H. (2019). Adult and infant pharmacokinetic profiling of dihydrocodeine using physiologically based pharmacokinetic modeling. Biopharm. Drug Dispos..

[213] Kovar L., Weber A., Zemlin M., Kohl Y., Bals R., Meibohm B., Selzer D., Lehr T. (2020). Physiologically-Based Pharmacokinetic (PBPK) Modeling Providing Insights into Fentanyl Pharmacokinetics in Adults and Pediatric Patients. Pharmaceutics.

[214] Emoto C., Fukuda T., Johnson T.N., Adams D.M., Vinks A.A. (2015). Development of a Pediatric Physiologically Based Pharmacokinetic Model for Sirolimus: Applying Principles of Growth and Maturation in Neonates and Infants. CPT Pharmacomet. Syst. Pharmacol..

[215] Walsh C., Bonner J.J., Johnson T.N., Neuhoff S., Ghazaly E.A., Gribben J.G., Boddy A.V., Veal G.J. (2016). Development of a physiologically based pharmacokinetic model of actinomycin D in children with cancer. Br. J. Clin. Pharmacol..

[216] Idkaidek N., Hamadi S., Bani-Domi R., Al-Adham I., Alsmadi M., Awaysheh F., Aqrabawi H., Al-Ghazawi A., Rabayah A. (2020). Saliva versus Plasma Therapeutic Drug Monitoring of Gentamicin in Jordanian Preterm Infants. Development of a Physiologically-Based Pharmacokinetic (PBPK) Model and Validation of Class II Drugs of Salivary Excretion Classification System. Drug Res..

[217] Mansoor N., Ahmed M., Czejka M., Sharib S., Hassan S., Hassan A. (2022). Pharmacokinetics of Midazolam in preterm neonates with an insight in brain Tissue: A PBPK approach. Pak. J. Pharm. Sci..

[218] Jiang X.L., Zhao P., Barrett J.S., Lesko L.J., Schmidt S. (2013). Application of physiologically based pharmacokinetic modeling to predict acetaminophen metabolism and pharmacokinetics in children. CPT Pharmacomet. Syst. Pharmacol..

[219] Zhao W., Le Guellec C., Benjamin D.K., Hope W.W., Bourgeois T., Watt K.M., van den Anker J.N., Matrot B., Saxen H., Hoppu K. (2014). First Dose in Neonates: Are Juvenile Mice, Adults and In Vitro-In Silico Data Predictive of Neonatal Pharmacokinetics of Fluconazole. Clin. Pharmacokinet..

[220] Xu R., Tang H., Chen L., Ge W., Yang J. (2021). Developing a physiologically based pharmacokinetic model of apixaban to predict scenarios of drug-drug interactions, renal impairment and paediatric populations. Br. J. Clin. Pharmacol..

[221] Donovan M.D., Abduljalil K., Cryan J.F., Boylan G.B., Griffin B.T. (2018). Application of a physiologically-based pharmacokinetic model for the prediction of bumetanide plasma and brain concentrations in the neonate. Biopharm. Drug Dispos..

[222] Olafuyi O., Abbasi M.Y., Allegaert K. (2021). Physiologically based pharmacokinetic modelling of acetaminophen in preterm neonates-The impact of metabolising enzyme ontogeny and reduced cardiac output. Biopharm. Drug Dispos..

[223] Zhang M., Yu Z., Liu H., Wang X., Li H., Yao X., Liu D. (2023). Model-informed drug development: The mechanistic HSK3486 physiologically based pharmacokinetic model informing dose decisions in clinical trials of specific populations. Biopharm. Drug Dispos..

[224] Bonner J.J., Burt H., Johnson T.N., Whitaker M.J., Porter J., Ross R.J. (2021). Development and verification of an endogenous PBPK model to inform hydrocortisone replacement dosing in children and adults with cortisol deficiency. Eur. J. Pharm. Sci..

[225] McGavin J.K., Goa K.L. (2001). Ganciclovir: An update of its use in the prevention of cytomegalovirus infection and disease in transplant recipients. Drugs.

[226] Duan P., Wu F., Moore J.N., Fisher J., Crentsil V., Gonzalez D., Zhang L., Burckart G.J., Wang J. (2019). Assessing CYP2C19 Ontogeny in Neonates and Infants Using Physiologically Based Pharmacokinetic Models: Impact of Enzyme Maturation Versus Inhibition. CPT Pharmacomet. Syst. Pharmacol..

[227] Abduljalil K., Jamei M., Rostami-Hodjegan A., Johnson T.N. (2014). Changes in individual drug-independent system parameters during virtual paediatric pharmacokinetic trials: Introducing time-varying physiology into a paediatric PBPK model. AAPS J..

[228] Salem F., Small B.G., Johnson T.N. (2022). Development and application of a pediatric mechanistic kidney model. CPT Pharmacomet. Syst. Pharmacol..

[229] Pan X., Stader F., Abduljalil K., Gill K.L., Johnson T.N., Gardner I., Jamei M. (2020). Development and Application of a Physiologically-Based Pharmacokinetic Model to Predict the Pharmacokinetics of Therapeutic Proteins from Full-term Neonates to Adolescents. AAPS J..

[230] Edginton A.N., Schmitt W., Willmann S. (2006). Development and evaluation of a generic physiologically based pharmacokinetic model for children. Clin. Pharmacokinet..

[231] van der Heijden J.E.M., Freriksen J.J.M., de Hoop-Sommen M.A., van Bussel L.P.M., Driessen S.H.P., Orlebeke A.E.M., Verscheijden L.F.M., Greupink R., de Wildt S.N. (2022). Feasibility of a Pragmatic PBPK Modeling Approach: Towards Model-Informed Dosing in Pediatric Clinical Care. Clin. Pharmacokinet..

[232] Duan P., Fisher J.W., Yoshida K., Zhang L., Burckart G.J., Wang J. (2017). Physiologically Based Pharmacokinetic Prediction of Linezolid and Emtricitabine in Neonates and Infants. Clin. Pharmacokinet..

[233] Abduljalil K., Pan X., Pansari A., Jamei M., Johnson T.N. (2020). Preterm Physiologically Based Pharmacokinetic Model. Part II: Applications of the Model to Predict Drug Pharmacokinetics in the Preterm Population. Clin. Pharmacokinet..

[234] Ince I., Dallmann A., Frechen S., Coboeken K., Niederalt C., Wendl T., Block M., Meyer M., Eissing T., Burghaus R. (2021). Predictive Performance of Physiology-Based Pharmacokinetic Dose Estimates for Pediatric Trials: Evaluation with 10 Bayer Small-Molecule Compounds in Children. J. Clin. Pharmacol..

[235] Rajput A.J., Aldibani H.K.A., Rostami-Hodjegan A. (2023). In-depth analysis of patterns in selection of different physiologically based pharmacokinetic modeling tools: Part I—Applications and rationale behind the use of open source-code software. Biopharm. Drug Dispos..

[236] Aldibani H.K.A., Rajput A.J., Rostami-Hodjegan A. (2023). In-depth analysis of patterns in selection of different physiologically-based pharmacokinetic modeling tools: Part II—Assessment of model reusability and comparison between open and non-open source-code software. Biopharm. Drug Dispos..

[237] El-Khateeb E., Burkhill S., Murby S., Amirat H., Rostami-Hodjegan A., Ahmad A. (2021). Physiological-based pharmacokinetic modeling trends in pharmaceutical drug development over the last 20-years; in-depth analysis of applications, organizations, and platforms. Biopharm. Drug Dispos..

[238] T’Jollyn H., Vermeulen A., Van Bocxlaer J. (2019). PBPK and its Virtual Populations: The Impact of Physiology on Pediatric Pharmacokinetic Predictions of Tramadol. AAPS J..

[239] Dinh T.H., Delaney K.P., Goga A., Jackson D., Lombard C., Woldesenbet S., Mogashoa M., Pillay Y., Shaffer N. (2015). Impact of Maternal HIV Seroconversion during Pregnancy on Early Mother to Child Transmission of HIV (MTCT) Measured at 4–8 Weeks Postpartum in South Africa 2011–2012: A National Population-Based Evaluation. PLoS ONE.

[240] Fischl M.A., Richman D.D., Grieco M.H., Gottlieb M.S., Volberding P.A., Laskin O.L., Leedom J.M., Groopman J.E., Mildvan D., Schooley R.T. (1987). The efficacy of azidothymidine (AZT) in the treatment of patients with AIDS and AIDS-related complex. A double-blind, placebo-controlled trial. N. Engl. J. Med..

[241] Zhuravel S.V., Khmelnitskiy O.K., Burlaka O.O., Gritsan A.I., Goloshchekin B.M., Kim S., Hong K.Y. (2021). Nafamostat in hospitalized patients with moderate to severe COVID-19 pneumonia: A randomised Phase II clinical trial. EClinicalMedicine.

[242] van Donge T., Pfister M., Bielicki J., Csajka C., Rodieux F., van den Anker J., Fuchs A. (2018). Quantitative Analysis of Gentamicin Exposure in Neonates and Infants Calls into Question Its Current Dosing Recommendations. Antimicrob. Agents Chemother..

[243] Dallos P., Wang C.Y. (1974). Bioelectric correlates of kanamycin intoxication. Audiology.

[244] Ryan A., McGee T.J. (1977). Development of hearing loss in kanamycin treated chinchillas. Ann. Otol. Rhinol. Laryngol..

[245] Huth M.E., Ricci A.J., Cheng A.G. (2011). Mechanisms of aminoglycoside ototoxicity and targets of hair cell protection. Int. J. Otolaryngol..

[246] Contrepois A., Brion N., Garaud J.J., Faurisson F., Delatour F., Levy J.C., Deybach J.C., Carbon C. (1985). Renal disposition of gentamicin, dibekacin, tobramycin, netilmicin, and amikacin in humans. Antimicrob. Agents Chemother..

[247] Smith P.B., Cohen-Wolkowiez M., Castro L.M., Poindexter B., Bidegain M., Weitkamp J.H., Schelonka R.L., Ward R.M., Wade K., Valencia G. (2011). Population pharmacokinetics of meropenem in plasma and cerebrospinal fluid of infants with suspected or complicated intra-abdominal infections. Pediatr. Infect. Dis. J..

[248] Lim S.Y., Miller J.L. (2022). Ampicillin Dose for Early and Late-Onset Group B Streptococcal Disease in Neonates. Am. J. Perinatol..

[249] Ecker K.L., Donohue P.K., Kim K.S., Shepard J.A., Aucott S.W. (2013). The impact of group B Streptococcus prophylaxis on early onset neonatal infections. J. Neonatal Perinat. Med..

[250] Castagnola E., Jacqz-Aigrain E., Kaguelidou F., Maragliano R., Stronati M., Rizzollo S., Farina D., Manzoni P. (2012). Fluconazole use and safety in the nursery. Early Hum. Dev..

[251] Tamimi J.J., Salem I.I., Alam S.M., Zaman Q., Dham R. (2005). Bioequivalence evaluation of two brands of lisinopril tablets (Lisotec and Zestril) in healthy human volunteers. Biopharm. Drug Dispos..

[252] Hirota K., Yoshioka H., Kabara S., Kudo T., Ishihara H., Matsuki A. (2001). A comparison of the relaxant effects of olprinone and aminophylline on methacholine-induced bronchoconstriction in dogs. Anesth. Analg..

[253] Armanian A.M., Badiee Z., Afghari R., Salehimehr N., Hassanzade A., Sheikhzadeh S., Sharif Tehrani M., Rezvan G. (2014). Prophylactic aminophylline for prevention of apnea at higher-risk preterm neonates. Iran. Red Crescent Med. J..

[254] Maharaj A.R., Edginton A.N., Fotaki N. (2016). Assessment of Age-Related Changes in Pediatric Gastrointestinal Solubility. Pharm. Res..

